# Transportation-related factors, cancer screening, and stage at diagnosis: A scoping review

**DOI:** 10.1371/journal.pone.0354856

**Published:** 2026-08-10

**Authors:** Alexa L. Pohl, Leora A. Cohen-Tigör, Aderinsola A. Aderonmu, Alexander Doan, Lee Horton, Bona Ko, Blynn Shideler, Arden M. Morris

**Affiliations:** 1 S-SPIRE Center, Department of Surgery, Stanford University School of Medicine, Stanford, California, United States of America; 2 Dell Medical School at the University of Texas at Austin, Austin, Texas, United States of America; 3 Duke University School of Medicine, Durham, North Carolina, United States of America; Dayeh University, TAIWAN

## Abstract

**Background:**

Over 4 million Americans annually miss healthcare appointments – including for cancer screening and treatment – due to transportation insecurity. We performed a scoping review to understand how transportation affects cancer screening and stage at diagnosis, and to make recommendations for measuring transportation-related factors in this context.

**Methods:**

We searched PubMed, TRID, Embase, Scopus, and Web of Science for full-length, English-language, quantitative observational studies published from January 1, 2003 to June 12, 2025. We included observational studies on screening or stage measuring transportation barriers quantitatively at the patient level. Two authors reviewed content, extracted data, and assessed quality. We compared methods of measuring transportation-related factors and associations between these factors and screening/stage at diagnosis.

**Results:**

We screened 4567 abstracts, reviewed 446 full papers, and identified 53 screening and 43 stage at diagnosis studies after quality assessment. Transportation exposures were classified into *travel distance/time*, *vehicle access*, *public transit*, *needing help to travel*, *patient perception of distance/time*, and *patient perception of transportation access*. Travel distance/time accounted for 74% of analyses, and results were mixed between worse cancer outcomes for farther travelers (47%) and null or reversed results, but these findings were moderated by rurality and sociodemographic status. Owning a vehicle and being able to travel independently were associated with better clinical outcomes. Per quality assessment, papers were susceptible to referral and information bias; additionally, spatial auto-correlation and the hierarchical nature of data were frequently ignored leading to inflated effects.

**Conclusions:**

Future research should go beyond “travel distance” to include alternative measures like vehicle ownership, car-less episodes, and needing help to travel to holistically evaluate transportation as a social driver of health.

## Introduction

Cancer patients who experience barriers to transportation have higher rates of ER utilization and cancer-specific mortality than those with ready access to transportation [1]. However, it is unclear whether these disparate outcomes are due to advanced stage at diagnosis or missed treatments or both [2], with implications for both preventive and therapeutic care. Using national survey data, we recently found that barriers to transportation were associated with forgoing screening for breast cancer, but not colorectal or cervical cancer [3]. Such differences between cancer types could be due to the additional trip required by breast cancer screening. Alternatively, those eligible for breast cancer screening – women 40–74 years of age – could be more vulnerable to social determinants of health [4], specifically *transportation-related factors* which in combination determine *transportation insecurity*, defined as the inability to reliably and safely travel at predictable times [5].

A more comprehensive understanding of the impact of transportation-related factors on cancer screening and stage at diagnosis is needed to inform potential interventions to address transportation insecurity in cancer screening. Objective measures *(e.g., vehicle ownership)* – which we term *transportation exposures* – are in many cases only proxies for specific transportation-related factors *(e.g., vehicle access)*; however, these exposures are frequently presented in isolation, without a conceptual framework, interfering with the ability of researchers and policymakers alike to draw clear implications. Current literature on transportation and cancer detection commonly uses researcher-calculated time or distance to medical facilities as the exposure. Unfortunately, while intuitive and simple to collect, driving distance is not experienced equally, as patients’ willingness to travel is influenced by their socioeconomic status and social context [6–8]. For example, affluent patients seeking high quality care may bypass local hospitals, traveling further as a result, while patients with few resources may forgo care if long-distance travel is required [9]. Travel distance also does not account for the safety, accessibility, or reliability of cars or public transportation, nor for needing another’s help to make the journey. Alternative measures of transportation exposures – including personal vehicle ownership, travel time on public transportation, and needing another’s help to make the journey – are subject to similar risks of conflating exposures and factors, but are also needed to build a comprehensive picture of transportation insecurity and cancer detection.

It is unclear to what extent the literature on transportation and cancer detection reflects the multidimensional experience of transportation insecurity, which includes lateness, skipping trips, social isolation, and worrying due to transportation problems, as well as time burden [10]. Therefore, we conducted a scoping review to identify studies on transportation and cancer detection (including *screening/surveillance* and *stage at diagnosis*) to answer the following questions: *How are transportation-related factors measured? Is transportation insecurity associated with non-adherence to screening and/or advanced stage at diagnosis? What bias is present related to the measurement of transportation insecurity?* and *How should transportation insecurity be measured going forward?*

## Methods

### Framework

Our theoretical framework, Andersen’s behavioral model of health service utilization, classifies individual and contextual factors linked to health behaviors as predisposing factors, enabling factors, and determinants of perceived need [11]. Under this model, the decision to engage in a health-related behavior – like cancer screening – is determined by interaction between individual and community socioeconomic status and health beliefs (*predisposing factors*), tangible individual and community resources (*enabling factors*), and filtered through an individual’s perception of their own health status, based on symptoms and personal health information (*perceived need*). Transportation is both an individual and contextual enabling factor, as an individual’s ability to get from place to place is determined by their own access, and by the transportation infrastructure of their community.

### Search strategy

We searched the published literature using PubMed, TRID, Embase, Scopus, and Web of Science databases to locate studies that assessed the relationship between transportation and cancer-related outcomes, including (i) screening adherence and (ii) stage at diagnosis. Specific search terms were developed with the assistance of a reference librarian. Search strings for all databases are found in Supplemental Table 1 and also at https://github.com/S-SPIRE-Research; the PubMed search terms are included here as an example:

(cancer*[tiab] OR neoplasms[majr]) AND (“screening”[tiab] OR “early detection of cancer”[mh] OR “treatment”[tiab] OR “therapy”[sh] OR “therapeutics”[mh] OR “aftercare”[mh] OR follow-up[tiab] OR surveillance[tiab] OR “chemoradiotherapy”[MeSH Terms] OR “chemoradiotherapy”[tiab] OR “chemoradiation”[tiab] OR “stage diagnosis”[tiab: 2] OR “neoplasm staging”[MeSH] OR “cancer stag*”[tiab] OR “radiotherapy”[Subheading] OR “radiotherapy”[tiab] OR “radiation therapy”[tiab] OR “radiotherapy”[MeSH Terms] OR “surgery”[Subheading] OR “surgery”[tiab] OR “postoperative care”[Mesh]) AND (“transportation”[MeSH] OR “transportation of patients”[MeSH] OR “driving time”[tiab: 15] OR “driving distance”[tiab: 15] OR “distance treatment”[tiab: 2] OR “travel time”[tiab: 15] OR “traveled time”[tiab: 15] OR “travel distance”[tiab: 15] OR “traveled distance”[tiab: 15] OR “transportation barrier*”[tiab] OR “travel burden”[tiab] OR “distance from hospital*”[tiab] OR (“health services accessibility”[mh] AND “transportation”[tiab])) NOT (“aviation”[MeSH] OR “ships”[MeSH] OR “railroads”[MeSH] OR “occupational diseases”[MeSH]).

No gray literature was included. According to best practices, we submitted the protocol for this scoping review in PROSPERO but our protocol was rejected by an automatic agent because it was identified by the agent as a scoping review, which were not supported by PROSPERO as the time of submission; key elements of the study protocol can be found in Supplemental Table 2, Supplemental Table 3 and Supplemental Table 4.

Only full-length English-language articles published after 2003 were eligible for inclusion. The starting date of 2003 reflected publication of the Institue of Medicine’s white paper *Unequal Treatment* [12], which provided a new conceptual framework for social determinates of health, as well as increased digitalization of measurement more translatable to current practice. We included case-control, cross-sectional, and cohort studies that measured transportation barriers and reported a quantitative cancer-related outcome from individual patient data. No qualitative studies, systematic reviews, or meta-analyses were considered. We excluded clinical trials or experimental studies where transportation security was manipulated but we did include secondary data analysis of clinical trials of an unrelated treatment. Studies published until June 12th, 2025 were screened for inclusion. Transportation-related barriers could be measured as either an individual categorical, ordinal, or continuous variable, or as a categorical, ordinal, or continuous constructed latent variable. Eligible study subjects were adults  > 18 years of age but otherwise no restrictions were placed on nationality, gender, or age of the participants.

### Manuscript screening

We used Covidence (Veritas Health Innovation, Melbourne, Australia) to organize manuscript screening and data extraction. Two authors independently reviewed each title and abstract for eligibility. In the case of disagreement at this stage of review, we defaulted to keeping the article for full-text review. Two authors independently reviewed each full-length article to determine eligibility for final inclusion. Disagreements were discussed together with the senior author, who adjudicated if consensus was not achieved.

### Data extraction

Two co-authors independently extracted data from the included studies. Variables included year of publication, country, study design, primary cancer outcome, cancer type(s), population, study dates, data source, database used, and sample size. For each transportation-related variable, two co-authors extracted the variable name, how the variable was calculated, and categorized the exposure. The adjusted effect sizes, confidence intervals, and contrasts for each transportation-related predictor variable were extracted for each primary and secondary cancer outcome. Finally, we recorded the overall results of the study and any key concerns as free text. The extracted results were checked for consistency and discussed within the study team until consensus was achieved.

### Quality assessment

Publications were assessed for quality by two authors using the Johanna Briggs Institute critical appraisal tools for cohort, case-control, or cross-sectional studies [13]. The tools contain questions in the domains of selection and allocation, comprehensiveness of reporting, classification of exposure, confounding factors, temporal precedence (cohort only), outcome measurement, participant retention (cohort only), and statistical conclusion validity (Supplemental Table 4). Two authors recorded specific concerns as free text. When threats to quality and rigor were considered too extensive to include in the scoping review (e.g., no statistical adjustment of confounders) by at least two study team members, papers were presented to the senior author and inclusion vs exclusion was discussed to consensus. *No statistical adjustment of confounders* was defined by the use of univariate analyses (e.g., Chi-Squared test); we were lenient and accepted any reasonable attempt at controlling for confounders appropriate to the study design and population. No specific confounder was considered essential given the sex- and age- specific nature of cancer screening.

### Study comparison

We categorized measurements into the following categories during data extraction, based on preliminary reading of the first 100 abstracts for full review: *Access to vehicle, travel time, route distance, point-to-point distance, missing appointments, and other*. After data extraction from all papers for full review, we revisited the categorization of transportation variables and two authors discussed to consensus, with special attention to the “Other” category and potential need to expand or collapse categories, emulating a hybrid coding approach. Ultimately, we collapsed travel time, route distance, and point-to-point distance together into “driving distance/time”, retained vehicle access, eliminated missing appointments, and split the other category into public transit accessibility, needing help to travel, patient perception of distance/time as a barrier, and patient perception of transportation access as a barrier. As some studies included more than one type of cancer, outcome, or transportation variable, analyses were grouped by exposure, outcome and cancer type, and each group was counted to summarize the distribution of the literature. Analyses that only differed slightly (e.g., using different methods to calculate driving distance) were combined. As meta-analysis was not feasible due to heterogeneity in outcomes and measurement, comparisons are discussed in a narrative format, with emphasis on studies exceeding n = 100,000 participants. Free-text extraction and quality assessment data were reviewed to generate a list of potential sources of bias; this list was narrowed to 7 methodological issues specific to measuring transportation insecurity in the context of cancer detection.

## Results

**Fig 1:**
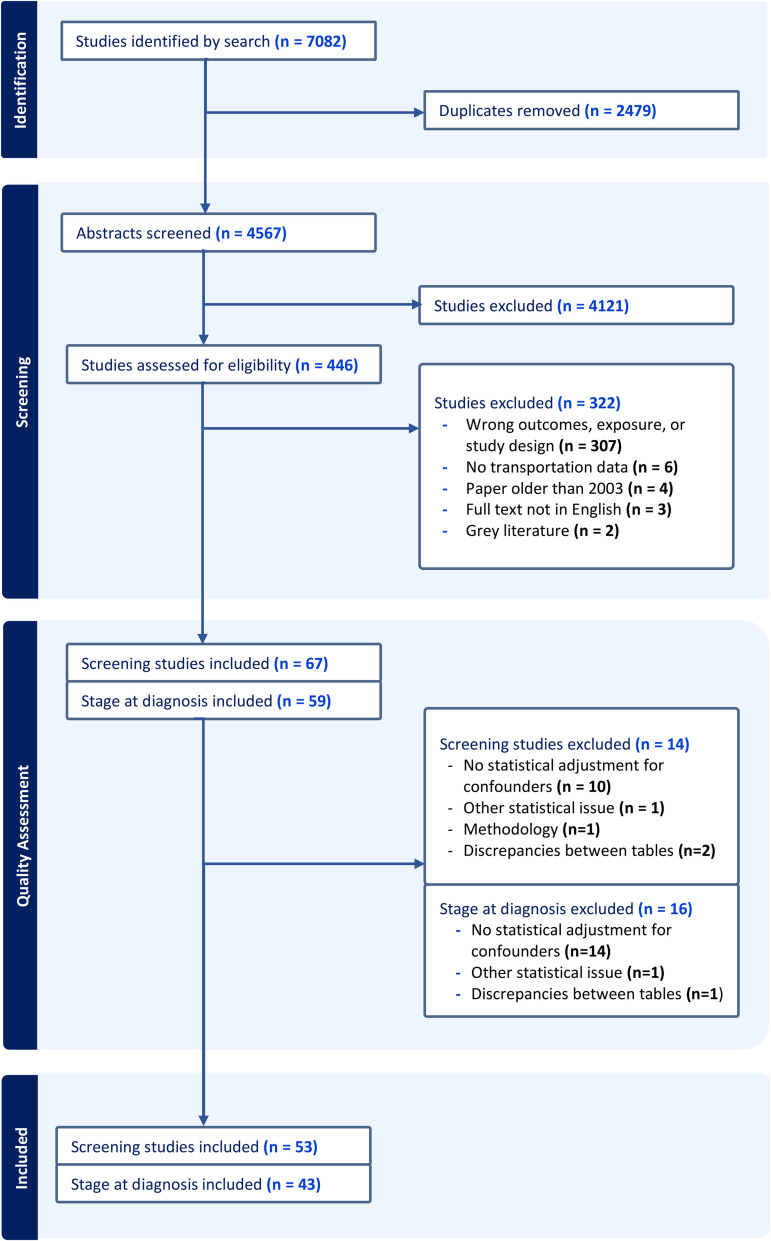
PRISMA diagram.

Our literature search identified 4567 unique publications, of which 446 underwent full text review. Among these, 124 studies met criteria for inclusion. Studies were tagged by outcome variable(s); 67 and 59 studies were identified with screening and stage at diagnosis as outcomes, respectively. After data extraction and additional exclusions (14 screening [10 for no statistical adjustment for confounders, 1 for other statistical issue, 1 for methodology issue, 2 for discrepancies between tables], 16 stage at diagnosis [14 for for no statistical adjustment for confounders, 1 for other statistical issue, 1 for discrepancies between tables]), we were left with a total of 53 screening and 43 stage at diagnosis studies (PRISMA diagram, Fig 1). There were 4 pairs of studies that re-used the same data [14,15–17,18,19,20,21], and these studies were combined for analysis; two pairs of studies had partially overlapping data [22,23,24,25] and were analyzed separately.

Transportation exposures were classified into six categories: Driving distance/time, vehicle access, public transportation accessibility, needing help to travel, patient perception of distance/time as a barrier, and patient perception of transportation access as a barrier (Table 1). Driving distance/time was the most common transportation-related exposure studied (56% of screening analyses, 94% of stage-at-diagnosis analyses). Patient perception of transportation access as a barrier, patient perception of travel time as a barrier, and needing the help of another to travel were only studied in the context of cancer screening.

**Table 1:** Distribution of analyses by cancer outcome, cancer type, and transportation variable.

Transportation Variable	Cancer Type	Screening	Stage at Diagnosis
Driving distance/time	Breast	15	20
	Cervical	7	3
	Colorectal	7	10
	Lung	4	5
	Other	6	24
Access to vehicle	Breast	3	1
	Other		1
Public transit accessibility	Breast	2	1
	Colorectal	2	
	Lung	1	
	Other		1
Needs help of another for transportation	Breast	1	
	Cervical	1	
	Colorectal	1	
Perceived Barrier – distance/time	Breast	3	
	Cervical	1	
Perceived barrier – transportation access	Breast	5	
	Cervical	4	
	Colorectal	5	
	Lung	1	
	Other	1	
**Total**		**70**	**66**

### Driving distance/time

We found substantial heterogeneity among the studies that used driving distance/time as an exposure: over 15 types of cancer were studied across screening and stage at diagnosis in nearly 20 countries, with exposures measured in kilometers, miles, minutes, and percentiles, and effects reported as odds ratios, hazards ratios, relative risk ratios, regression coefficients, and marginal probability effects (Table 2, Table 3, Supplemental Table 5, Supplemental Table 6, Supplemental Table 7, Supplemental Table 8, Supplemental Table 9, Supplemental Table 10, Supplemental Table 11, Supplemental Table 12, Supplemental Table 13, Supplemental Table 14).

**Table 2:** Findings from studies of transportation insecurity and breast cancer screening.

Study	Outcome	Population	N	Exposure	Results (95% CI)	Summary
Addo 2023 [26]	Lifetime clinical breast exam	Women 15–49 in DHS data from (Burkina Faso, Côte d’Ivoire, Kenya, Lesotho, Namibia)	45,945	Self-reported perception of problem posed by travel distance	Travel not a problem: aOR **1.30 (1.21–1.39)**	Those who did not find distance “a big problem” were more likely to have been previously screened for breast cancer.
Ali 2025 [27]	Lifetime clinical breast exam	Kenyan women 15–49	10,267	Mode of transport	Vehicle: aOR **1.34 (1.12–1.62)**	Using a vehicle to access healthcare was associated with increased use of clinical breast exam.
Ayebeng 2025 [28]	Lifetime clinical breast exam	Women 25–49 in DHS data from (Burkina Faso, Côte d’Ivoire, Ghana, Kenya, Mozambique, Senegal & Tanzania)	65,486	Self-reported perception of problem posed by distance or by traveling alone	Distance barrier: aOR **0.95 (0.89–0.99)** Traveling alone barrier: **aOR 1.19 (1.10–1.28)**	Reporting distance as a barrier was associated with reduced use of breast exam. Reporting traveling alone as a barrier was associated with increased use of breast exam.
Banegas 2024 [29]	Screening mammography w/in 2 years	US Women 50–74 eligible for breast cancer screening in 186 community-based healthcare organizations	83,993	Patient-reported transportation barriers	Barriers: **aRR 0.94 (0.90–0.98)**	Patients reporting transportation barriers were less likely to be up to date with screening.
Dao 2023 [30]	Time interval between diagnostic imaging and biopsy (BI-RADS 4–5)	Women undergoing diagnostic mammography between 2015–2020 at Boston Medical Center	2,885	Trouble getting transportation to medical appointments	No significant findings	No association between transportation issues and time from diagnostic mammography to biopsy.
Giordano 2012 [31]	Mammogram at 1 year after recieving invitation	Women 40–45 living in Turin, Italy	5,649	Bus travel time	≤ 20 min: **aOR 1.67 (1.45–1.92)** 20−30 min: **aOR 1.19 (1.04–1.37)**	Shorter bus travel time increased mammography use compared to trips ≥ 40 min.
Giuliani 2016 [32]	Using yearly mammography or clinical breast exam	Women diagnosed with *in situ* or invasive breast cancer on the Romagna Italy Cancer Registry from 1990–2000	1,304	Travel time from patient’s home to nearest hospital with mammography	≥ 30 min: **aOR 0.44 (0.29–0.68)**	Travel time ≥ 30 minutes associated with lower odds of yearly surveillance vs ≤ 15 min.
Henry 2014 [33]	Mammogram w/in 2 years	Women 40–74 living in Utah	5,197	Travel time to nearest mammography center	No significant findings	No relationship between travel time and mammogram use.
Jensen 2014 [34] ^1^	Participation in screening mammogram after invitation in a national health care system	Women 50–69 living in Denmark	127,628	Driving distance from house to assigned screening site (ref < 15km)	15−25 km: aRR **0.90 (0.85–0.95)** 25−35 km: aRR **0.86 (0.81–0.91)** 35−45 km: aRR **0.77 (0.71–0.83)** 45−55 km: aRR **0.74 (0.68–0.80)** 55−65 km: aRR **0.77 (0.71–0.83)** > 65 km: aRR **0.74 (0.66–0.82)**	Mammogramy use negatively associated with distance from screening site; relationship exacerbated in women without vehicle access.
Jewett 2018 [35]	Reciept of at least one mammogram every 2 years	Women 50–74 in Wisconsin with no history of breast cancer	5,930	Driving time from participant’s home to nearest mammography site	20 vs 10 min (ref): aOR **1.56 (1.10–2.23)** 40 vs 30 min (ref): aOR **0.70 (0.51–0.96)**	No relationship between driving distance and mammography uptake in urban participants. Non-linear effects in rural participants: doubling time from 10 to 20 min associated with increased uptake, but 30–40 min associated with decreased uptake.
Khan 2021 [36]	Proportion of women receiving a mammogram w/in 6 months of screening invitation	Women 50–69 invited to particpate in state-wide breast cancer screening in Sydney, Australia	227,474 women in 9,528 census tracts	% car ownership (census tract) Co-location (Bus stop / train station) Distance from centroid to nearest mammography center, weighted for opening hours	% Car ownership: Reg. coeff. **1.42 (1.17–1.68)** Co-location (Bus): Reg. coeff. 0.39 (−0.04–0.81) Co-location (Train): Reg. coeff. **−1.89 (- 2.38 - −1.40)** Distance (2^nd^ quartile): Reg. coeff. **1.82 (1.14–2.51)** Distance (3^rd^ quartile): reg. coeff **3.90 (3.12–4.58)** Distance (4^th^ quartile): Reg. coeff. **6.25 (5.49–7.01)**	Increasing car ownership rates to 1 SD above the mean was associated with a near doubling of the proportion of women receiving a mammogram. Venue co-location with a train station was negatively associated with mammogram uptake. Increasing distance from the nearest screening center was significantly associated with *increased* screening rates with a dose-dependent effect.
Khang 2017 [37]	Diagnostic workup completion	Low-income women 47–67 with BI-RADS 4–5 living in South Carolina between 1996–2009	1,073	Route distance to diagnosing mammography facility	< 5 mi: aHR **1.41 (1.00–1.80)** 5–10 mi: aHR **1.33 (1.03–1.72)**	Distances < 10 miles positively associated with workup completion vs > 15 mi.
Lairson 2005 [38]	Screening mammogram w/in 15 months	US Female Veterans 52+	3,415	Self-reported travel time to last mammography appointment	No significant findings	No relationship between travel time and mammography use.
Maheswaran 2006 [39]	Reciept of screening mammogram after invitation	Women 50–64 invited for breast cancer screening in North Derbyshire, UK	34,868	Route distance from patient’s home to assigned screening location	aOR **0.87 (0.79–0.95)**	Route distance negatively associated with screening attendance.
Miller 2021 [40]	Return for annual mammogram during COVID	Women with screening mammogram 1 year prior to COVID disruption in the Univ. VA Health System	10,213	Driving time from patient’s residence to screening facility	aOR **0.88 (0.84–0.91)**	Increased travel time negatively associated with returning for mammogram during COVID-19.
Mobley 2009 [41]	Mammography claim code w/in 1 year	California women 65 + enrolled in Medicare A&B between 2002–2003	70,129	Distance / Commuter Intensity (proportion of workers communiting > 60 min. to work)	Distance: MPE **−0.001 (p < 0.05)** Commuter Intensity: MPE **−0.14 (p < 0.05)**	Likelihood of mammogram decreased 0.1% per mile above mean distance. Commuter intensity decreased probability by 14%. Disabled women were *more* likely to be screened if they lived in a commuter-intense neighborhood.
Onega 2011 [42]	Surveillance mammography between 6–18 mo. and 18–30 mo. after completion of primary therapy.	Women with Stage I/II breast cancer recieving care through Group Health in Washington state.	1,012	Travel time from patient residence to nearest Group Health mammogram facility.	No significant findings	No relationship between receipt of surveillance mammography and distance.
Onitilo 2013 [43]	Any screening mammogram in 5 years prior to breast cancer diagnosis	Women with breast cancer in a single health care system in Wisconson	1,368	Travel time from patient residence to nearest mammography facility	aOR **0.99 (0.99–0.99)**	Negative association between time to facility and receipt of screening in 5 years prior to diagnosis.
Pohl 2025 [3]	Patient-reported mammogram w/in 2 years	US Women 50–74	5,823	Delayed medical care in past due to transportation barriers	Reported barriers aOR **0.59 (0.40–0.86)**	Women experiencing transportation barriers were less likely to be up-to-date.
Rollet 2021 [15] & Rollet 2023 [16]	Mammogram w/in 2 years of invitation	10% sample of women 50–74 invited to participate in national breast cancer screening in Frane, 2013–2014	397,598	Travel time from residence to nearest mammography facility	Time: aOR **0.94 (0.93–0.95)** Age x Time: aOR **0.99 (0.98–1.00)**	Screening decreased with travel time; largest effect on elderly women. Variation by region.
Sedani 2025 [17] & Miller [14]^1^	Screening mammogram w/in 2 years	US Women 40–74 (2022 BRFSS)	54,506	Lack of reliable transport	Transportation insecurity aRR **0.82 (0.77–0.87)**	Lacking reliable transportation associated with lower likelihood of being up-to-date.
St-Jacques 2013 [44]	Screening mammogram w/in 2 years	Women 52–69 in Quebec during 2008	833,856	Route distance from patient’s residence to nearest facility (ref < 12.5km)	12.5−25 km: aRR **0.96 (0.96–0.97)** 25−50 km: aRR **0.96 (0.95–0.97)** 50−75 km: aRR **0.88 (0.86–0.89)** > 75 km: aRR **0.81 (0.79–0.83)**	Screening mammography use associated with distance; patients in rural areas more affected.
Vyas 2014 [45]	Mammogram w/in 2 years	Women in West Virginia with 1 or more mammogram in past 10 years	1,104	Delayed care due to transport	Delayed care aOR **0.27 (0.08–0.89)**	Delaying care due to transportation negatively associated with adherence.
Wilson 2011 [46] ^1^	Screen during 3-yr interval	American-Indian women enrolled in Indian Health Service from 2004–2006	654	Route distance from patient ZIP code to nearest mammography facility	1−14 mi: aOR **0.34 (0.14–0.83)**	Living 1–14 miles away associated with decreased uptake vs living in the same town.

^1^Results reversed for ease of interpretation aOR adjusted odds ratio, aRR adjusted risk ratio, MPE marginal probability effect

**Table 3:** Findings from studies of transportation insecurity and stage at diagnosis in breast cancer.

Study	Outcome	Population	Sample	Exposure	Results (95% CI)	Summary
Bhangdia 2025 [47]	Stage at diagnosis	Women with breast cancer presenting at a Rwandan referral hospital between 2012–2016	426	Travel time	4th Quartile: aOR **2.46 (1.21–5.12)**	Higher stage at diagnosis was significantly associated with travel times within the 4th quartile.
Celaya 2010 [48]	Stage at Diagnosis	All women aged 40 and older living in New Hampshire, diagnosed with breast cancer between 1998–2004	5,833	Route distance/travel time from patient’s address to the nearest mammography facility	No significant results	Neither driving distance to the nearest mammography center nor the travel time to the nearest mammography center predicted stage at diagnosis in breast cancer.
deAlmeida 2022 [49]	Stage at diagnosis	Women aged 18 and older living in Sao Paolo and diagnosed with primary breast cancer between 2000–2015	81,669	Did patient have to travel to different municipality for treatment?	Different municipality: aOR **1.07 (1.04–1.10)**	Having to travel to a different municipality for cancer treatment was positively associated with presentation at a higher stage at diagnosis.
Dickens 2014 [50]	Stage at diagnosis	Women diagnosed with breast cancer at a tertiary referral center in South Africa from 2006–2012	1,071	Straight line distance from patient’s home to tertiary referral center	20−30 km: aRR **1.31 (1.08–1.57)** 30−40 km: aRR **1.40 (1.12–1.75)**	Probability of late stage at diagnosis significantly increased as distance increased from referral center through 40 km; after 40 km there was a plateau or perhaps even a reversal effect indicating a non-linear trend.
Gaba 2023 [51]	Stage at diagnosis	American Indian/Alaska Native and Non-Hispanic White women diagnosed with breast cancer from 2004–2016	809,125	Point to point distance	21−50 mi: aOR **1.17 (1.00–1.38**)	Decreasing travel distance was significantly associated with an increased odds of having Stage I breast cancer at diagnosis in both American Indian/Alaska Native women and Non-Hispanic White women.
Henry 2011 [19] & Henry 2013 [18]	Stage at diagnosis	Women 40 + diagnosed with breast cancer between 2004–2006 in population-based cancer registries in 10 US states	161,619	Driving time to diagnosing facility	10−20 min: aOR **0.95 (0.92–0.97**) 20−30 min: aOR **0.96 (0.92–0.99)** 40−50 min: aOR **0.83 (0.78–0.89)** > 60 min: aOR **0.88 (0.82–0.94)**	Longer travel times to diagnosing facility, but not nearest mammography facility, were associated with decreased stage at diagnosis. In a stratified analysis by state, travel time to nearest facility was associated with *increased* stage at diagnosis in Iowa only.
Huang 2009 [52]	Stage at diagnosis	Women aged 40 and older living in Kentucky with primary breast cancer diagnosed between 1999–2003	12,322	Route distance between patient and nearest mammography facility	>15 mi: aOR **1.50 (1.25–1.80)**	Women living greater than 15 miles from their nearest mammography facility had 50% higher odds of being diagnosed with locally advanced or metastatic breast cancer compared to those living within 5 miles.
Kim 2013 [53]	Abnormal screening mammogram result	Female patients residing in Cook County, IL who had received 1 or more mammograms and were living below 250% of the federal poverty level	21,085	Point to point distance from participant’s home address to the clinic where the participant was screened.	Distance (mi): Reg. coef. **1.06 (NR, p < 0.01)**	Distance was significantly associated with abnormal mammogram results in women living below 250% of the federal poverty level in Chicago, IL.
Knapp 2021 [54]	Stage at Diagnosis	Adults treated for primary breast cancer at Obafemi Awolowo University Teaching Hospital in Nigeria between 2009–2019	609	Travel time	3rd quintile: aOR **2.25 (1.09–4.63)** 4th quintile: aOR **2.53 (1.24–5.17)** 5th quintile: aOR **2.82 (1.30–6.11)**	Increasing travel time was associated with increased odds of late state at diagnosis. This effect persisted when the analysis was limited to patients residing in the expected catchment area of the hospital.
Lian 2012 [55]	Stage at diagnosis	Women diagnosed with breast cancer in St. Louis between 2002–2006	4,205	Driving time to nearest mammography facility	No significant results.	No relationship between travel time and stage at diagnosis.
Murage 2018 [56]	Diagnosis by emergent presentation	Patients diagnosed with primary breast cancer in several cancer registries in England between 2006–2010	183,400	Travel time in minutes to GP	10−20 min: aRR **1.18 (1.09–1.28)** 20−30 min: aRR **1.45 (1.18–1.79)**	Patients had significantly higher odds of diagnosis with an emergent presentation as travel time to GP increased.
Onega 2011 [42]	Stage at diagnosis	Adult women with a primary diagnosis of Stage I, IIA, or IIB breast cancer between 1990–1999 receiving care through Group Health in Washington State	1,012	Travel time from patient census block centroid to nearest network mammogram facility	No significant results	No significant relationship between travel time to nearest mammography facility and stage at diagnosis. This result was invariant to how stage at diagnosis was defined.
Schroen 2009 [57]	Stage at diagnosis	Women aged 40 and older diagnosed with primary breast cancer in Virginia between 2000–2001	8,170	Route distance	No significant results	No relationship between route distance and invasive tumor size at diagnosis when adjusting for patient age, race, and per capita income.
Scoggins 2012 [58]	Stage at diagnosis	Adults aged 18–64 enrolled in Medicaid in Washington State and diagnosed with breast cancer between 1997–2003	1,407	Travel time between patient’s and PCP’s zip code	Travel time (per 1 hr): aOR **1.27 (NR)** Route distance (per 100 mi): aOR **1.49 (NR)**	Increasing travel time/distance traveled was significantly associated with an increased stage at diagnosis in breast cancer.
Stapleton 2011 [59]	Stage at diagnosis	Adult women diagnosed with primary breast cancer between 2007–2008 at National Cancer Institute of Cairo University and Tanta Cancer Center in Egypt	343	Patient-reported travel time	No significant results	On univariate analysis, the late-stage group had longer average travel times, but the effect of travel time was not statistically significant in a multivariate model.
Tarlov 2009 [60]	Stage at diagnosis	Non-Hispanic White, Non-Hispanic African American, and Hispanic women aged 45 and older, living in Chicago and diagnosed with breast cancer between 1996–1998	4,533	Average route distance to 5 nearest mammography facilities Number of public transportation stops within a 0.25 mile radius of each facility	No significant results	Neither distance to nearest 5 mammography facilities or the number of public transit stops near the mammography facility impacted patient stage at diagnosis.
Tesfaw 2021 [61]	Stage at diagnosis	Breast cancer patients treated at Felege Hiwot and Gondar University hospitals in Ethiopia from 2019–2020	371	Patient-reported distance to hospital	>5 km: aOR **3.20 (1.72–5.29)**	Living > 5 km from the hospital was significantly associated with a 3-times increased odds of late stage at diagnosis.
Togawa 2021 [62]	Stage at diagnosis	Women aged 18 + diagnosed with breast cancer between 2014–2017 at regional, state, or national referral hospitals in four Sub-Saharan African countries	1,541	Straight-line distance to a diagnostic/treatment facility Mode of transit	aOR **1.04 (1.01–1.06)** No significant results.	Distance was associated with increased odds of late stage at diagnosis, but there was no association between stage at diagnosis and mode of transit.
Virgilsen 2019 [63]	Stage at diagnosis	Patients in the Danish Cancer Register (DCR) diagnosed with breast cancer between 2005–2016	3,758	Route distance between patient and their PCP Route distance between patient and diagnosing facility	No significant results	Neither distance to GP nor diagnosing hospital was significantly associated with advanced breast cancer.
Wang 2008 [20] & Wang 2010 [21]	Stage at diagnosis	Women diagnosed with breast cancer between 1998–2000 in Illinois	30,511	Driving distance/time to nearest mammography facility	No significant results	Driving distance or time to the nearest mammography facility was not associated with stage at diagnosis; result was unchanged when stratified by rural/urban context.

aOR adjusted odds ratio, aRR adjusted risk ratio, NR not reported

Across diverse populations and settings, greater travel distance/time to screening facilities were generally associated with lower uptake of breast cancer screening (mammography and clinical breast exams) and with delays in follow-up or diagnostic pathways. Four exemplar studies tested whether driving distance/time had a negative effect on breast cancer screening using records from governments with universal health care with sample sizes exceeding 100,000 participants [15,34,36,44]. Distance was negatively associated with screening in Quebec [44], central Denmark [34], and France [15], but was positively associated with screening in Sydney [36]. The same studies offer an explanation for this discrepancy: the effect of distance was moderated by socioeconomic and geographic variables including age [15], vehicle access [34], administrative district [15,44] and rurality [44]. Accordingly, a handful of smaller studies of distance and breast cancer screening that found no effects, [30,33,38,42] but the majority found that increased distance was associated with decreased screening (Table 2) [26,31,32,35,39,40,41,43,46].

Studies that examined associations between barriers to travel and screening for other cancer types, specifically cervical, colorectal, lung, and others, also found mixed results with modest inverse associations between travel distance/time and uptake of screening (Supplemental Table 5, Supplemental Table 6, Supplemental Table 7 and 206Supplemental Table 8). A single study of large magnitude found a negative association between travel distance and screening for cervical, colorectal, and skin cancer [64]. Results were mixed for smaller studies of cervical cancer screening, with 3 reporting null results, [46,65,66] 3 reporting decreased screening with distance [67,68,69], and one study reporting increased screening with increasing distance (Supplemental Table 5) [70]. Similarly, smaller colorectal cancer screening studies found decreased screening with distance [71,72,73] or null results (Supplemental Table 6) [65,66,74,75,76]. There were only 4 small studies on driving distance/time and lung cancer screening: 3 studies on vulnerable populations found no association between driving distance/time and screening [22,23,77], while a study of similar size in South Carolina did (Supplemental Table 7) [78].

In contrast to uptake of screening, greater travel distance/time was inconsistently associated with breast cancer stage at diagnosis (Table 3). There were 3 large studies on distance and stage at diagnosis in breast cancer [51,18,56]. Stage at diagnosis was increased with distance to PCP in a UK population registry [56], and stage at diagnosis was increased with distance to reporting hospital in a US hospital-based registry [51]. In contrast, stage at diagnosis appeared to be decreased with distance to diagnosing facility and showed no association with distance to mammography centers in a US population registry study spanning 10 states [19]. Further analysis suggested that the association between further distance and lower stage was confounded by area deprivation, and the association between distance and stage was moderated by state, with increasing distance linked to increased stage in Iowa only [18]. The use of different facilities – primary care, mammography, diagnosing hospital, and treating hospital – as reference points among these three studies makes it difficult to compare results. In addition to these large studies, 10 smaller studies reported increasing stage at diagnosis with increasing travel distance [47,49–51,52–54,58,61,62] and 7 reported null results (Table 3) [42,48,55,57,59,60,20].

There were 3 large studies on colorectal cancer stage at diagnosis. Two studies using overlapping data from the National Cancer Database in the US found an association between increasing distance from the reporting facility and metastatic disease [24,25]. Distance to PCP was also associated with increased stage at diagnosis in colorectal cancer in the UK [56] and Denmark, albeit with a smaller sample size. [63] The remainder of colorectal stage-at-diagnosis studies involving travel distance were all smaller with null results (Supplemental Table 10) [58,79,80,81]. Beyond breast and colorectal cancer, there were 5 or fewer studies on travel distance/time and stage at diagnosis per cancer type and there was no consistent association between transportation and cancer stage.

### Vehicle ownership, public transit, and needing another’s help to travel

Two large (n > 100,000) studies with data from national healthcare systems found that vehicle ownership was associated with higher rates of breast cancer screening [34,36] and that vehicle ownership moderated the association between travel distance and screening [34]. A smaller (n = 10,000) study of Kenyan women found that access to a private vehicle was associated with increased breast cancer screening [27], but there was no association between stage at diagnosis and mode of transportation among 1,000 breast cancer patients in four sub-Saharan countries. Lower stage at diagnosis was associated with vehicle ownership in prostate cancer [82]. Public transportation accessibility (e.g., number of nearby stops, transit travel time, % commuting by public transportation) was not associated with cancer screening or stage at diagnosis in 4 of 6 studies [31,36,74,76,77,60,82]. Needing the help of another to travel was negatively associated with cancer screenings in two of only three studies that explored this exposure [28,67,83].

### Patient perception: Distance/time and transportation accessibility as barriers to care

The perception of time/distance as a barrier to healthcare was negatively associated with breast cancer screening in an African multi-national study with over 45,000 participants [26], and this finding was recapitulated in a partially overlapping sample of over 60,000 participants two years later [28]. A single-nation study of roughly 6500 participants using the same dataset found null results for cervical cancer screening [84]. We classified an analysis that linked commute intensity with decreased breast cancer screening in over 70,000 California Medicare recipients [41] as patient perspective of time/distance as barrier, but recognize that areas of intense traffic may pose barriers other than increased time. Intriguingly, this study found a strong association between communities with higher proportion of elderly women living alone and mammography use. Finally, patient perception of transportation accessibility as a barrier was used as an exposure in 10 studies, with 7 of these studies published in the past 3 years [3,14,17,29,30,85,86]. Several US studies with national data found that participants reporting transportation access as a barrier were less likely to be up to date with breast cancer screening [3,17,29], but only the largest of the three studies found the same relationship for colorectal and cervical cancer screening [29]. Separately, a study of patients insured through a large US HMO found no relationship between severe transportation insecurity and colorectal cancer screening [85]. The remaining studies were small with niche populations [30,45,87,86,88].

### Synthesis of findings by cancer type

For breast cancer, 11 of 15 studies reported an inverse relationship between travel distance/time and screening [15,32,34,35,37,39,40,41,43,44,46]. 3 of 3 studies reported a positive relationship between vehicle access and screening [27,34,36], and 1 of 2 studies reported a positive relationship between public transit accessibility and screening [31]. A perceived barrier of distance/time was negatively associated with screening in 3 of 3 studies [26,28,41], and a perceived barrier of transportation access was negatively associated with screening in 4 of 5 studies [3,17,29,45]. A single study found needing another’s help to travel was paradoxically positively associated with breast cancer screening [28]. 11 of 20 studies found a positive relationship between travel distance/time and advanced stage at diagnosis in breast cancer [47,49–51,52,54,56,58,61], and a single study each found no relationship between vehicle access [62] or public transit accessibility [60] and advanced stage at diagnosis.

For cervical cancer, 4 of 7 studies reported either a null [46,65,66] or paradoxical relationship [70] between travel distance/time and screening, and 3 of 4 studies reported no relationship between perceived transportation access and cervical cancer screening. [3,17,87]. Needing another’s help to travel was negatively associated with cervical cancer screening in a single study [67], but perceived distance/time barriers were not, also in a single study [84]. 2 of 3 studies also reported no relationship between travel distance/time and stage at diagnosis in cervical cancer [56,63].

For colorectal cancer, 4 of 7 studies reported a negative association between travel distance/time and screening [64,71,72,73], and 3 of 5 studies reported a negative association between perceived transportation access and colorectal cancer screening [17,29,86]. 2 of 2 studies reported no relationship between public transit accessibility and colorectal cancer screening [74,76]; a single study reported a negative relationship between needing a relative to help with travel and colorectal cancer screening [83]. 6 of 10 analyses found no relationship between travel distance/time and stage at diagnosis in colorectal cancer [79]. (colon and rectal) [58,63 (colon only) 80,81].

For lung cancer, 3 of 4 studies found no relationship between travel distance/time and receipt of screening [22,23,77], but 3 of 5 studies found an positive relationship between travel distance/time and advanced stage at diagnosis [56,63,89]. In single studies, there was no relationship between public transit accessibility or perceived transportation accessibility and lung cancer screening [17,77].

### Risk of bias

**Fig 2:**
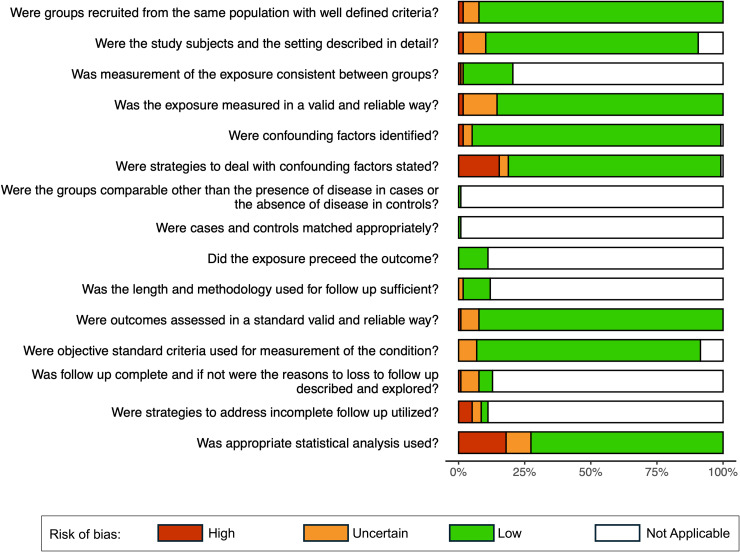
Critical appraisal of retained studies. Assessment of 118 papers on cancer screening and stage at diagnosis and transportation using the Joanna Briggs Institute tools for cross-sectional, cohort, and case-control studies. Some studies included both screening and stage at diagnosis as endpoints. Questions are paraphrased for brevity; full text can be found in Supplemental Table 4. Full assessments for each study are presented in Supplemental Figures 1-12 in S1 File.

Risk of bias emerged in repeated patterns within the domains of selection and allocation, classification of exposure, comprehensiveness of reporting, participant retention, confounding factors, and statistical conclusion validity (Fig 2,Supplemental Figure 1, Supplemental Figure 2, Supplemental Figure 3, Supplemental Figure 4 Supplemental Figure 5, Supplemental Figure 6, Supplemental Figure 7, Supplemental Figure 8, Supplemental Figure 9, Supplemental Figure 10, Supplemental Figure 11, Supplemental Figure 12). Several single-institution studies of stage at diagnosis were judged to be at high risk for referral bias [42,50,54,59,61,90,91]. Some authors argued that single-institution registries were *de facto* population registries in locations that have no other oncology referral centers [50,54,61,90] and others tried to limit referral bias by restricting the radius of the sample population [50,91].

Risk of bias related to participant retention and/or outcome (for cohort and cross-sectional studies respectively) was an issue for studies on cancer screening. In multiple studies, the outcome was defined as a record of cancer screening within the EMR during a pre-specified period, but strategies to detect and mitigate the effects of patients moving out of the health network, presenting with symptomatic cancers not detected by screening, and dying were not mentioned [22,23,30,40,42,43,46,53,78,92]. Studies that omitted tests for statistical interaction were at risk of bias, as over half of studies that tested for moderation of the relationship between transportation and cancer screening found significant effects [15,16,18,21,34,41,44,55,66,70,72,86,93]. Similarly, 6 of 10 studies that looked for non-linear relationships between travel burden and cancer screening found them [15,16,23,24,34,35,38,50,79,90,91].

Almost all studies controlled for age and one or more socioeconomic confounders (e.g., race/ethnicity, income, insurance status, education or marital status), but far fewer were able to control for the effect of patient comorbidities. Direct measurement of confounders was not always possible, particularly in anonymized databases created from medical records, so socioeconomic confounders were often assigned by patient geography (e.g., census tract, zip code). When a dataset is derived from a few geographic units surrounding a single facility, the distance measured may be related to landscape features or other place-based factors. Separating the effects of actual distance from place-based factors may present challenges of multicollinearity and risk that nearby people may have artificially similar outcomes (spatial auto-correlation). Similarly problematically, a few studies assigned exposures by patient geography rather than measuring distance or time [41,94,95]. This method created statistical problems when confounders and exposures were fixed effects at the geographical level but were treated as random effects in the model (i.e., a mixed-level model was not used) – such studies were not retained for full text analysis [95,96].

## Discussion

In this scoping review of transportation-related factors and cancer detection, over 55% of articles that fulfilled inclusion criteria on cancer screening and over 90% of similar articles on stage at diagnosis measured transportation related-factors by calculating the driving distance or time from the patient’s residence to a healthcare facility. Additional facets of transportation included *vehicle access, accessibility of public transportation* (including nearby stations and transit time), *needing another’s help with transportation, patients’ own perception of time/distance* and *availability of transportation as barriers to care*. We note that in most instances study teams necessarily measured only transportation exposures rather than the broader construct of transportation insecurity, which would require substantially more resources. Taken together, these papers indicate the association between driving distance/time and cancer screening is complex, likely contextual, moderated by external variables, and possibly non-linear. Overall, studies were at risk of bias when single-institution hospital registries were used as data sources, when patient attrition was not considered in annualized screening studies derived from EMR data, when the possibility of moderation or non-linear effects were ignored, and when statistical methods did not account for the spatial or clustered nature of the data.

Prior systematic reviews on transportation and cancer detection have focused on travel distance and geographic accessibility rather than on the multidimensional aspects of transportation insecurity [2,97,98]. Khan-Gates *et al.* [98] and Conti *et al.* [97] reviewed the breast cancer and spatial accessibility literature in 2013 and 2019 respectively, while Ambroggi *et al.* reviewed stage at diagnosis and travel burden across all cancers in 2014 [2]. Kahn-Gates *et al.* included 2 studies on mammography use and distance, 7 studies on stage and diagnosis and distance, and 8 studies on stage at diagnosis and travel time, and were unable to draw strong conclusions on a relationship between spatial accessibility and breast cancer detection [98]. Our scoping review included 11 of these 17 studies [99–103]. Inclusion of the additional papers would have increased the number of studies with null results for breast cancer stage at diagnosis, and thereby strengthened our conclusion that breast cancer and driving distance/time are overrepresented in the literature. A separate review found 2 additional studies [104,105] and 7 studies that overlapped with our search, and reported that patients traveling farther tended towards a higher stage at diagnosis [2].

With respect to Conti *et al.*, we included 2 overlapping studies [33,44] assessing mammography and spatial accessibility, and found an additional 11 studies on mammography uptake and driving time/distance published prior to 2019, as well as 3 studies published after. Based on this work, the authors recommended that distance should be measured relative to actual facilities used, that further work on public transit accessibility is needed, and that stratified or moderation analyses should be used to allow for the effect of distance to vary by urban/rural status or by socioeconomic status. Similarly, our findings indicate that distance commonly has a negative impact on screening uptake, but that the relationship is likely non-linear and moderated by other socioeconomic factors. The relationship between travel burden and stage at diagnosis remains unclear.

Vehicle ownership was associated with increased screening or earlier stage at diagnosis in breast and prostate cancer in 4 of 5 studies that met inclusion critiera [27,34,36,82]. As three of these studies were specific to breast cancer screening, these data generate the hypothesis that car ownership may be independently associated with breast cancer screening, but this hypothesis has yet to be tested in a large US-based population sample.

After reviewing common causes of bias in the relevant literature, we generated strategies for their mitigation, summarized as Table 4. Referral bias may create an artificial negative relationship between stage at diagnosis and distance to treating facility when participants are drawn from cancer registries maintained by tertiary hospitals. Using population-based samples and measuring distance to where the patient initially sought care could help to address this bias. We also acknowledge that referral bias may be subtle within hospital-based registries. For example, the National Cancer Database (NCDB) records about 70% of incident cancers in the US, but only a third of US hospitals contribute cases (i.e., those with American College of Surgeons Commission on Cancer accreditation) [106]; comparisons with SEER demonstrate that the NCDB has a different distribution of stage at diagnosis [107]. In the NCDB, when a patient is reported by multiple facilities, the most complete record is kept and others are discarded, and a summary of whether the patient was diagnosed and treated at the same facility is produced [108]. Analyses using the NCDB to study travel distance and stage at diagnosis should report whether their cohort is restricted to patients diagnosed at the reporting facility, as in Massarweh et al. [24].

**Table 4:** Methodological challenges to studying transportation insecurity as a social driver of health.

Concept	Definition	Example	Potential Implications	Mitigation
Referral Bias	Patients referred to tertiary care have more advanced or rarer conditions	A tertiary care center will treat patients with advanced disease from a large catchment area, but will only treat local patients with early disease.	Studies derived only from tertiary care centers will overestimate the relationship between distance and stage at diagnosis.	Use population registries as data source; consider if diagnosing or treating facility should be used; stratify by disease severity.
Information Bias	Misclassification of exposure, confounder, or outcome status	A study using presence/absence of screening in the EMR may misclassify patients who obtained screening out-of-network.	Urban patients may have more screening sites to choose from, leading to spurious relationships between rurality and screening.	Use outcome data not tied to a single system (e.g., insurance claims); limit EMR use to closed networks (e.g., HMOs).
Moderation	A third variable changes the relationship between exposure and outcome	Patients with fewer resources find long travel distances a deterrent, but distance may not affect those with more resources.	Risk of overlooking strong effects of travel burden on cancer outcomes among socioeconomically vulnerable persons.	Pre-specify stratified analysis where empiric evidence exists; test for interaction in multivariable models.
Linearity Bias	Assumption of a consistent proportional relationship between exposure and outcome	The negative impact of travel time may plateau if most patients are unwilling to travel more than (e.g.,) an hour.	Underestimation of relationship between transportation and cancer outcome due to poor model fit.	Plot exposure vs outcome using continuous data to detect non-linear effects prior to analysis.
Fixed Effects	Exposure or confounding variable is assigned based on group membership	Vehicle access is assigned based on the percent of households that own a vehicle within the participant’s county.	Failure to account for fixed effects leads to underestimation of standard errors (Type I errors).	Use multi-level models when data is clustered and independence of observations is violated.
Spatial Auto-correlation	An exposure, confounding or outcome variable is spatially distributed	Minoritized racial/ethnic groups are clustered in neighborhoods due to historical policies (e.g., redlining).	Underestimation of standard errors leads to overestimation of statistical significance (Type I errors).	Report Moran’s I and use spatial lag models when spatial auto-correlation is significant.
Multi-collinearity	A variable is collinear with distance due to its spatial distribution	A hospital is located within a wealthy neighborhood, so patient income decreases with distance from the hospital.	Model will struggle to differentiate the effect of distance from the effect of income.	Report the variance inflation factor (VIF). Avoid datasets where distance is measured relative to a single location.

Many studies calculated annualized screening rates based on EMR data in lieu of performing a time-to-event analysis for cancer screening [40,42,76,109,110], which could introduce bias related to unrealized attrition. Urban residents may have more choices for where to obtain cancer screening and not all may be captured by a single institution’s EMR, whereas rural residents may realistically only have access to a single facility. For cancer screening outcomes, this bias could be mitigated by restricting the use of the EMR as a data source to health care networks where patients are unlikely to access care elsewhere (e.g. HMOs). Over half of the studies that tested for moderation effects or non-linear relationships found them, suggesting that assuming a linear and unmoderated relationship between travel burden and cancer outcomes would bias the field towards null results.

Spatial autocorrelation reduces the effective sample size of observations of variables that are organized geographically, and the bias introduced is sufficient to obscure true relationships between social determinants and health outcomes [111]. Spatial autocorrelation should be accounted for in an analysis of any variable that is spatially distributed (e.g., wealth, neighborhood deprivation, mortality). The extent of spatial autocorrelation in cancer registries is difficult to assess as the geographic data of individual patients is typically withheld for privacy. A sensible approach would be to test for spatial autocorrelation when residential location information is available to the researcher.

We also note that using spatially autocorrelated variables to predict one another creates multicollinearity. Travel distance is inherently spatially distributed; especially when assessed relative to an individual facility. In this situation, travel distance will be multicollinear with any other variable (e.g., income) that is structured in space, and the effect of distance will be impossible to partition from other social determinants of health. Assigning variation in cancer outcomes to travel distance may only be possible in datasets where patients can reasonably be assumed to be independently distributed observations, and there is a negligible relationship between distance and other socioeconomic variables.

In addition to these statistical issues with travel distance/time, there are numerous conceptual issues as well. First, researcher-calculated travel time underestimates the travel burden of racially minoritized groups, which leads to an underestimation of the negative effects of transportation barriers on cancer outcomes [112]. Second, most researchers measure the distance or travel time between the participants’ home address and treating facility, but the presence of health care facilities within a person’s typical activity space (the geographic footprint where they work, shop for groceries, etc.) – rather than distance to a doctor per se – predicts healthcare utilization [113]. Finally, patients choose healthcare providers based on a number of attributes, including perceived quality, waiting times, and travel distance/time, and will weigh attributes based on their socioeconomic context [114,115]. Patients are willing to travel for higher quality care, including patients living in poverty [116]; however, this behavior is exceedingly difficult to model, as patients are willing to travel for better outcomes in hypothetical situations, but favor nearby facilities in reality [114].

Findings varied by the type of transportation measure used. Studies relying on objective distance or travel time often showed weaker, non-linear, or context-dependent associations that were amplified in rural or low-resource settings, whereas studies incorporating self-reported barriers or transportation access tended to show stronger and more consistent associations with screening non-adherence and later-stage diagnoses. This pattern suggests that perceptual and experiential dimensions of transportation insecurity capture additional burden not reflected by distance alone. Multilevel or interaction-focused designs that model distance alongside vehicle access, socioeconomic status, and rurality often reveal moderation and non-linear patterns that simpler models miss, underscoring the inadequacy of distance as a sole proxy for transportation-related factors.

Given the methodological issues with travel distance/time, complementary ways to measure transportation barriers and novel sources of data are needed to better understand transportation as a social determinant of health. We recommend basing further study on a multi-domain framework that can be measured across studies. Such a framework would ideally include affordability (costs of travel, car ownership and maintenance, insurance, out-of-pocket expenses; financial subsidies as potential mitigators), reliability (availability and punctuality of transportation modes, trip cancellations, wait times, consistency of access), safety (perceived and actual safety of travel environments, crime and traffic risk, weather-related hazards), autonomy (ability to travel independently without reliance on others, need for assistance or caregiver involvement, ability to schedule and complete trips without undue coordination burdens), and time burden (total travel time, waiting time, number of trips, opportunity costs such as missed work).

Accordingly, *private vehicle ownership* is one promising alternative – it is objective, and is already tracked as an economic indicato. [117]. The *ratio of drivers to vehicles in a household* may be a more sensitive measure, as it will differentiate between those with no access, limited access, and unlimited access to a vehicle [118]. As car ownership is precarious for low-income households, the *duration and number of car-less episodes* should be characterized in relation to health outcomes, particularly as 40% of respondents acquiring a car after a car-less episode cited attending medical appoints as a motivating factor in their decision [119]. The applicability of *public transit accessibility* is uncertain, given that individuals with transportation barriers overwhelmingly rely on being driven by others to medical appointments, rather than increasing their public transit use [4]. Data on *perceived transportation access* is increasingly available as universal screening for social determinants of health becomes mainstream – both Epic and Cerner include a single item on transportation insecurity in their out-of-the-box screening module [120]. Unfortunately these data will be subject to inherent limitations of EMR data, including representativeness and data completeness [121]. Ultimately, active data collection is needed to capture both the material and psychosocial aspects of transportation insecurity, and a validated Transportation Security Index is under development for this purpose [122].

## Limitations

Despite the use of standardized extraction and quality assessment instruments, all scoping reviews include investigator decision making. Because epidemiological studies are less protocolized than clinical trials, scoping reviews of epidemiological studies require more adjudication of edge cases, and our study is no exception. A strength of the current study is its inclusion of a true risk-of-bias assessment, rather than a comprehensiveness-of-reporting assessment [123]. While we acknowledge that the topical complexity inherent to transportation insecurity may have as much or more influence than methodological bias, a major goal for this project was to identify and offer recommendations for limiting bias. We used the JBI tools for bias assessment – while they are excellent at rapidly identifying studies that are irredeemably flawed, they are less sensitive to differences in quality between minimally valid studies. The Cochrane Risk-of-Bias in Non-randomized Studies of Exposure (ROBINS-E) tool is an alternative that captures bias more broadly; however, many authors hold that the ROBINS-E tool may not be practicable at this point in its development [124]. Additionally, aggregating screening and stage at diagnosis studies into a global risk-of-bias assessment may have obscured relationships between study design and sources of bias. Other strengths of the current study include consultation with a librarian to develop the search terms, and searching in non-medical indexes to capture work done by geographers and social scientists. We acknowledge that restricting to English-language-only introduces a potential bias, although we note that we purposefully sought global papers in order to avoid limiting conclusions to US or Western-based countries only.

## Conclusion

After retrieving over 4,500 publications, we identified 53 and 43 studies on transportation and cancer screening and stage at diagnosis, respectively. The vast majority considered driving distance/time as the transportation variable, but vehicle access, public transportation accessibility, needing help to travel, patient perception of distance/time as a barrier, and patient perception of transportation access as a barrier were also studied. Despite considerable efforts to quantify the effect of driving distance/time on cancer detection, no strong conclusions could be drawn. Indeed, we found the literature on transportation insecurity to be considerably limited by to an extent by bias but mostly by its inherent complexity.Therefore, we recommend considering complementary or alternative markers of transportation insecurity – especially vehicle access and patient perception of transportation access as a barrier to care. Standardizing use of such variables, especially in larger datasets, may reveal substantiative relationships that could, in turn, justify policy change to help patients who cannot drive themselves to preventative healthcare appointments.

To manage this complexity, we recommend operationalizing our proposed multi-dimensional framework through standardized variables in research that in turn may reveal important relationships with broader implications. For example, health systems could deploy standardized measurement of transportation insecurity in routine SDOH screenings, deploy patient navigators and telehealth-enabled scheduling to mitigate barriers, and collaborate with transportation planners to situate screening facilities where needs are greatest, with pilot programs rigorously evaluated for effects on screening adherence and stage at diagnosis to guide scale-up. Policy makers and payers could implement multi-dimensional transportation supports — such as vehicle access subsidies to address affordability and autonomy, rides to screening to address affordability and reliability, and co-located screening at community hubs to address safety, autonomy, and time burden — in the service of reducing travel burden and increasing cancer screening uptake in underserved and rural populations.

## Supporting information

S1 File Supplemental Figures and Tables [125–127,128–132,133–135,136–138]. (DOCX)

S2 File PRISMA 03 18. (PDF)
