# Supplementary material for: Transportation-related factors, cancer screening, and stage at diagnosis: A scoping review
Source: PLoS One. 2026 Aug 10;21(8):e0354856. doi: 10.1371/journal.pone.0354856 (PMC13456292; doi:10.1371/journal.pone.0354856)
Supplement: Supplementary file 1 [file pone.0354856.s002.pdf]

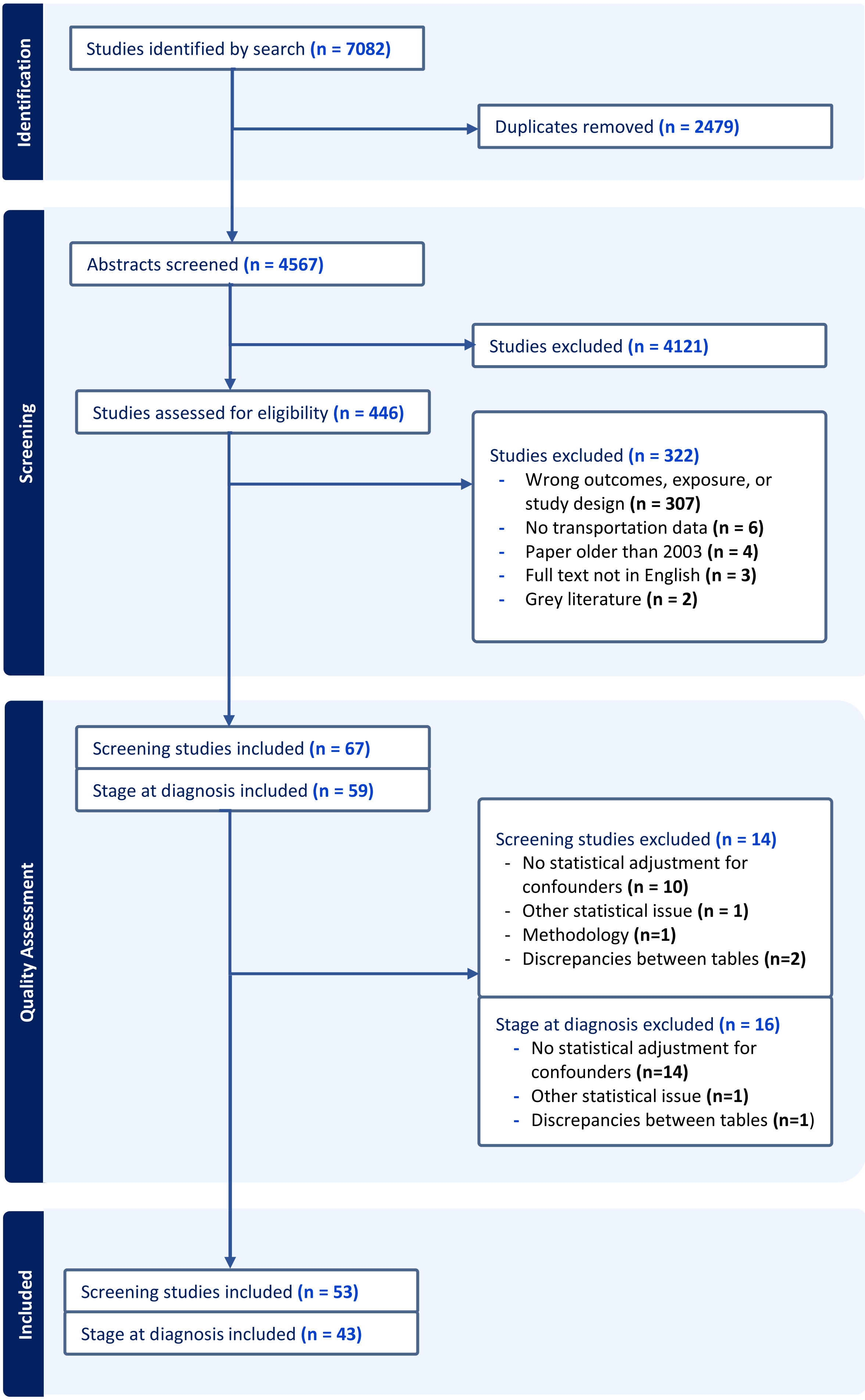
Identification
Studies identified by search (n = 7082)
Duplicates removed (n = 2479)
Screening
Abstracts screened (n = 4567)
Studies excluded (n = 4121)
Studies assessed for eligibility (n = 446)
Studies excluded (n = 322)
- Wrong outcomes, exposure, or study design (n = 307)
- No transportation data (n = 6)
- Paper older than 2003 (n = 4)
- Full text not in English (n = 3)
- Grey literature (n = 2)
Quality Assessment
Screening studies included (n = 67)
Stage at diagnosis included (n = 59)
Screening studies excluded (n = 14)
- No statistical adjustment for confounders (n = 10)
- Other statistical issue (n = 1)
- Methodology (n=1)
- Discrepancies between tables (n=2)
Stage at diagnosis excluded (n = 16)
- No statistical adjustment for confounders (n=14)
- Other statistical issue (n=1)
- Discrepancies between tables (n=1)
Included
Screening studies included (n = 53)
Stage at diagnosis included (n = 43)
