# Supplementary material for: Transportation-related factors, cancer screening, and stage at diagnosis: A scoping review
Source: PLoS One. 2026 Aug 10;21(8):e0354856. doi: 10.1371/journal.pone.0354856 (PMC13456292; doi:10.1371/journal.pone.0354856)
Supplement: Supplementary file 2 [file pone.0354856.s001.docx]

Supplementary Table 1. Search terms for each database

| Database | Search Terms |
| --- | --- |
| PubMed | (cancer*[tiab] OR neoplasms[majr]) AND ("screening"[tiab] OR "early detection of cancer"[mh] OR "treatment"[tiab] OR "therapy"[sh] OR "therapeutics"[mh] OR "aftercare"[mh] OR follow-up[tiab] OR surveillance[tiab] OR "chemoradiotherapy"[MeSH Terms] OR "chemoradiotherapy"[tiab] OR "chemoradiation"[tiab] OR "Stage Diagnosis"[tiab:~2] OR "neoplasm staging"[MeSH] OR "cancer stag*"[tiab] OR "radiotherapy"[Subheading] OR "radiotherapy"[tiab] OR "radiation therapy"[tiab] OR "radiotherapy"[MeSH Terms] OR "surgery"[Subheading] OR "surgery"[tiab] OR "Postoperative Care"[Mesh]) AND ("Transportation"[MeSH] OR "Transportation of Patients"[MeSH] OR "driving time"[tiab:~15] OR "driving distance"[tiab:~15] OR "Distance treatment"[tiab:~2] OR "travel time"[tiab:~15] OR "traveled time"[tiab:~15] OR "travel distance"[tiab:~15] OR "traveled distance"[tiab:~15] OR "transportation barrier*"[tiab] OR "Travel burden"[tiab] OR "distance from hospital*"[tiab] OR ("Health Services Accessibility"[mh] AND "transportation"[tiab])) NOT ("Aviation"[MeSH] OR "Ships"[MeSH] OR "Railroads"[MeSH] OR "Occupational Diseases"[MeSH]) |
| Scopus | ( TITLE-ABS-KEY ( ( cancer* OR neoplasms ) ) AND TITLE-ABS-KEY ( ( screening OR "early detection of cancer" OR treatment OR therapy OR therapeutics OR aftercare OR follow-up OR surveillance OR chemoradiotherapy OR chemoradiation OR "radiation therapy" OR radiotherapy OR “stage W/2 diagnosis” OR chemotherapy OR surg* OR "Postoperative Care" OR "Health Services Accessibility" ) ) AND TITLE-ABS-KEY ( ( "Transportation" OR "Transportation of Patients" OR "driving W/15 time" OR "driving W/15 distance" OR “travel W/15 distance” OR "travel W/15 time" OR "travel W/15 distance" OR "transportation barrier*" OR "Travel burden" OR "distance from hospital*" ) ) ) AND ( LIMIT-TO ( DOCTYPE , "ar" ) ) AND ( LIMIT-TO ( SUBJAREA , "MEDI" ) ) |
| Embase | (cancer*:ab,ti,kw OR 'neoplasm'/exp/mj) AND ('traffic and transport'/de OR 'travel'/de OR (driving NEAR/5 time) OR (driving NEAR/5 distance) OR (travel NEAR/5 time) OR (travel NEAR/5 distance) OR 'transportation barrier*':ab,ti,kw OR 'travel burden':ab,ti,kw OR 'distance from hospital*':ab,ti,kw) AND (screening:ab,ti,kw OR 'early cancer diagnosis'/exp OR 'treatment':ti OR 'cancer therapy'/exp OR 'aftercare'/exp OR 'follow up':ti OR surveillance:ti OR 'patient monitoring'/exp OR 'cancer radiotherapy'/exp OR 'chemoradiotherapy':ab,ti,kw OR 'chemoradiation':ab,ti,kw OR 'stage near/2 diagnosis' OR 'radiotherapy':ab,ti,kw OR 'radiation therapy':ab,ti,kw OR 'cancer chemotherapy'/exp OR 'surgery':ab,ti,kw OR 'postoperative care'/exp OR 'health care access'/exp) AND ('article'/it OR 'article in press'/it) NOT ('aviation'/exp OR 'ships'/exp OR 'railway'/exp OR 'occupational disease'/exp) |
| Web of Science | ((TS=((cancer* OR neoplasms) AND (screening OR "early detection of cancer" OR treatment OR therapy OR therapeutics OR aftercare OR follow-up OR surveillance OR chemoradiotherapy OR chemoradiation OR "radiation therapy" OR radiotherapy OR “stage NEAR/2 diagnosis” OR chemotherapy OR surg* OR "Postoperative Care" OR "Health Services Accessibility") AND ("Transportation" OR "Transportation of Patients" OR "driving NEAR/15 time" OR "driving NEAR/15 distance" OR "travel NEAR/15 time" OR "travel NEAR/15 distance" OR "transportation barrier*" OR "Travel burden" OR "distance from hospital*"))) AND (DT==("ARTICLE" OR "EARLY ACCESS"))) AND (SJ==("ONCOLOGY" OR "GENERAL INTERNAL MEDICINE" OR "HEALTH CARE SCIENCES SERVICES" OR "SURGERY" OR "OBSTETRICS GYNECOLOGY" OR "RADIOLOGY NUCLEAR MEDICINE MEDICAL IMAGING" OR "REHABILITATION" OR "HEMATOLOGY" OR "NURSING" OR "PEDIATRICS" OR "EDUCATION EDUCATIONAL RESEARCH" OR "ENDOCRINOLOGY METABOLISM" OR "IMMUNOLOGY" OR "OTORHINOLARYNGOLOGY" OR "UROLOGY NEPHROLOGY" OR "GERIATRICS GERONTOLOGY" OR "DERMATOLOGY" OR "CARDIOVASCULAR SYSTEM CARDIOLOGY" OR "OPHTHALMOLOGY" OR "TRANSPLANTATION" OR "URBAN STUDIES")) |
| Transport Research International Documentation (TRID) | (cancer* OR neoplasms) AND (screening OR "early detection of cancer" OR treatment OR therapy OR therapeutics OR aftercare OR follow-up OR surveillance OR chemoradiotherapy OR chemoradiation OR stage OR "radiation therapy" OR stage diagnosis” OR radiotherapy OR chemotherapy OR surg* OR "Postoperative Care" OR "Health Services Accessibility”) |

Supplemental Table 2. Inclusion/exclusion criteria used for title/abstract and full text review

|  | **Include** | **Exclude** |
| --- | --- | --- |
| **Population** | Adults > 18 years of age | Children  Institutionalized populations |
| **Exposure** | Transportation burden  Transportation cost  Travel distance  Travel time  Vehicle ownership  Vehicle access  Transportation insecurity  Travel-transportation covariate  Other transportation-related topics | No travel or transportation related data  High volume/high travel vs low volume/low travel*  Transportation as part of a composite variable* |
| **Outcome** | Screening Adherence  Surveillance Adherence  Stage at Diagnosis  Completion of screening (where screening is a two-step process, e.g. colonoscopy after FIT)*  Interval from screen to biopsy* | Measures of cost  Participation in clinical trials/interventions  Delay from diagnosis to treatment  Rates of follow-up care (excluding surveillance)  Distance to treatment center from zip code or census tract as outcome- (e.g. radiation facility allocation)* |
| **Study characteristics** | Observational Studies  Cohort Studies  Cross-Sectional Studies  Case-Control Studies  Secondary analysis of RCT data | Randomized controlled trials  Qualitative analyses  Systematic Reviews |
| **Other** |  | Articles not written in English  Articles published prior to 2003 (IoM Unequal Treatment/SDoH definition)  Discrete choice experiments* |
| * Denotes addition to study protocol during title/abstract and full text review to clarify common edge cases | | |

Supplemental Table 3. Data extraction template

|  | Instructions and multiple choice options (if applicable) |
| --- | --- |
| **Publication Identification** | |
| Year |  |
| DOI |  |
| Country | *Country in which the study was conducted. If multiple, list in other*  1. United States  2. UK  3. Canada  4. Australia  5. Other |
| **Methods** | |
| Cancer Outcome | *Check all that apply*  1. Screening  2. Stage at diagnosis |
| Cancer Type | *Check all that apply.*  1. Breast  2. Colorectal  3. Lung  4. Prostate  5. Other |
| Design | 1. Randomized controlled trial  2. Non-randomized experimental study  3. Cohort study  4. Cross sectional study  5. Case control study  6. Other |
| **Cohort definition** | |
| Population | *Brief description of study population; for example: "Randomly-selected national sample of American women 40 and older"* |
| Cohort dates | *Starting and ending year defining population in YYYY-YYYY format.* |
| Data source | 1. Single institution  2. Multiple institution consortium  3. State or national database  4. Other |
| Database name | *If the population is drawn from a national database, please provide the name; for example, "National Cancer Database".* |
| Sample Size |  |
| **Transportation Variables** | |
| Transportation Variable 1 | *How did the researchers measure transportation insecurity/barriers? Please chose the best match.*    *"Route distance" refers to distance measured using route mapping software, like arcGIS or Google Maps. "Point to point distance" is distance as the crow flies.*  1. Access to vehicle  2. Travel time  3. Route distance  4. Point to point distance  5. Missing appointments  6. Other |
| Trans Var 1 Method | *Briefly describe how the variable above was measured.* |
| Trans Var 2 | *If a second variable related to transportation was measured, please categorize it:*  1. Access to vehicle  2. Travel time  3. Route distance  4. Point to point distance  5. Missing appointments  6. Other |
| Trans Var 2 Method | *Briefly describe how variable above was measured* |
| **Cancer Outcome** | |
| Cancer Outcome Description | *Describe the specific cancer-related end-point; for example: "five year disease-free survival", "overall mortality", "patient-reported health-related quality of life".* |
| Cancer Outcome Effect Sizes | *"Contrast" means the reference group vs test group for the effect size being measured. For example, "0-1 cars(ref) vs 2+ cars".* *Effect size measure - Hazards Ratio, Prevalence Ratio, Odds Ratio, etc.* *If no effect size given but p-value given for comparison, describe result in "notes" below.* \| **Trans Var 1** \| **Trans Var 2**; **Name** \| \|; **Adj estimate** \| \|; **95% CI Adj estimate** \| \|; **Contrast** \| \|; **Effect size measure** \| \| |
| Cancer Outcome Confounders | *Which of these confounders did the authors adjust for in the results you recorded above? Check all that apply*  1. Age  2. Sex  3. Income  4. Race/ethnicity  5. Education  6. Marital status  7. Comorbidities  8. Disease Severity  9. Other |
| Cancer Outcome Notes | *If the directionality of the effect isn't clear from the table and results above, please elaborate:* |

Supplemental Table 4. Risk of bias assessment

| **Domain** | Cohort | Cross-Sectional | Case-Control |
| --- | --- | --- | --- |
| **Selection and allocation (1)**  *Yes/No/Uncertain* | Were the two groups similar and recruited from the same population? | Were the criteria for inclusion in the sample clearly defined? | Were the same criteria used for identification of cases and controls? |
| **Comprehensiveness of reporting (1)**  *Yes/No/Uncertain* | Not Applicable | Were the study subjects and the setting described in detail? | Not Applicable |
| **Classification of exposure (1)**  *Yes/No/Uncertain* | Were the exposures measured similarly to assign people to both the exposed and unexposed groups? | Not Applicable | Was exposure measured in the same way for cases and controls? |
| **Classification of exposure (2)**  *Yes/No/Uncertain* | Was the exposure measured in a valid and reliable way? | | |
| **Confounding factors (1)**  *Yes/No/Uncertain* | Were confounding factors identified? | | |
| **Confounding factors (2)**  *Yes/No/Uncertain* | Were strategies to deal with confounding factors stated? | | |
| **Confounding factors (3)**  *Yes/No/Uncertain* | Not Applicable | Not Applicable | Were the groups comparable other than the presence of disease in cases or the absence of disease in controls? |
| **Confounding factors (4)**  *Yes/No/Uncertain* | Not Applicable | Not Applicable | Were cases and controls matched appropriately? |
| **Temporal precedence (1)**  *Yes/No/Uncertain* | Were the groups/participants free of the outcome at the start of the study (or at the moment of the exposure)? | Not Applicable | Was the exposure period of interest long enough to be meaningful? |
| **Temporal precedence (2)**  *Yes/No/Uncertain* | Was the follow-up time reported and sufficient to be long enough for outcomes to occur? | Not Applicable | Not Applicable |
| **Assessment, detection, and measurement of the outcome (1)**  *Yes/No/Uncertain* | Were the outcomes measured in a valid and reliable way? | Were the outcomes measured in a valid and reliable way? | Were outcomes assessed in a standard, valid and reliable way for cases and controls? |
| **Assessment, detection, and measurement of the outcome (2)**  *Yes/No/Uncertain* | Not Applicable | Were objective, standard criteria used for measurement of the condition? | Not Applicable |
| **Participant retention (1)**  *Yes/No/Uncertain* | Was follow-up complete and, if not, were the reasons to loss to follow-up described and explored? | Not Applicable | Not Applicable |
| **Participant retention (2)**  *Yes/No/Uncertain* | Were strategies to address incomplete follow-up utilized? | Not Applicable | Not Applicable |
| **Statistical conclusion validity (1)***  *Yes/No/Uncertain* | Was appropriate statistical analysis used? | | |
| **Quality of statistical analysis***  *Excellent/Average/Poor* | Describe the quality of the statistical analysis performed. | | |
| **Overall assessment**  *Include/Exclude* |  | | |

*Reviewers were advised to answer the item appropriate for each particular study’s type. Free text justification could be included with any of the items.*

** We eliminated all studies that scored “No” on the question “Was appropriate statistical analysis used?”, leaving no way of distinguishing included studies further; therefore, we added an additional space to classify the quality of the statistical analysis into “Excellent”, “Average”, or “Poor”, as we felt there was additional granularity to be captured in this domain. For example, a study that failed to adjust for spatial autocorrelation but otherwise used appropriate analyses would be marked as appropriate on the JBI tool but would fail to meet the bar for an “Excellent” analysis on our quality assessment.*

Supplemental Table 5. Findings from studies of transportation insecurity and cervical cancer screening.

| **Study** | **Outcome** | **Population** | **Sample** | **Exposure** | **Results** | | | **Summary** |
| --- | --- | --- | --- | --- | --- | --- | --- | --- |
|  |  |  |  |  |  | aRR | 95% CI |  |
| Adams 2020 [39] | Cervical cancer screening within past year | Women aged 18-64 who utilized Federally Qualified Health Centers in South Carolina from 2007-2010 | 13709 | Route distance | 3rd quartile | **1.02** | **1.00-1.04** | Patients living *further* from the FQHC were significantly *more* likely to have had a cervical cancer screening in the past year at Health Centers 1 (n=13709) and 2 (n=5492), but not at Health Center 3 (n=6157). |
|  |  |  |  |  | 4th quartile | **1.02** | **1.00-1.03** |  |
|  |  |  | 5492 | Route distance | 2nd quartile | **1.02** | **1.00 - 1.05** |  |
|  |  |  |  |  | 4th quartile | **1.03** | **1.00 - 1.05** |  |
|  |  |  | 6157 | Route distance | *No significant results* | | |  |
|  |  |  |  |  |  | aOR | 95% CI |  |
| Andersson 2021 [40]^,A^ | Cervical cancer screening within past 10 years | Women 33-60 living in Stockholm who were HPV + or who had been treated for CIN2+ | 482 | Needed another person's help to attend gynecology appointment | Needed help | **0.14** | **0.07- 0.34** | Needing another person's help to attend the gynecological exam and travel times of > 60 minutes to the clinic were independently associated with decreased odds of cervical cancer screening in clinic. |
|  |  |  |  |  |  |  |  |  |
|  |  |  |  | Travel time | > 60 min | **0.25** | **0.09 – 0.68** |  |
| Aruma 2024 [41] | Self-reported Pap test within past three years | Convenience sample of women 45-65 living in Pennsylvania | 474 | Travel time to PCP | *No significant results* | | | Travel time to PCP was not associated with being up-to-date with cervical cancer screening |
|  |  |  |  |  |  | aRR | 95% CI |  |
| Banegas 2024 [17] | Pap test within past 3 years or pap + HPV co-test within past 5 years | Women eligible for cervical cancer screening in 186 community-based healthcare organizations | 202895 | Patient-reported transportation barriers | Barriers | **0.93** | **0.89 - 0.96** | Women reporting transportation barriers had a lower likelihood of being up-to-date with screening. |
| Coronado 2004 [42] | Pap test within past three years | Women 18-64 from a random sample of households stratified by percent Hispanic ethnicity within an agricultural region of Washington State between 1998-1999 | 767 | Patient-reported lack of transportation as a barrier to care | *No significant results* | | | No association between patients experiencing a lack of transportation and cervical cancer screening adherence. Results did not differ by ethnicity or acculturation levels. |
|  |  |  |  |  |  | aOR | 95% CI |  |
| Ezechi 2014 [43] ^A^ | Attending follow-up appointment after positive screening test | Nigerian women who screened positive on direct visual inspection of the cervix in 2011-2012 | 108 | Distance from patient's home to gynecology clinic | > 10 km | **0.50** | **0.24 – 1.0** | Women living further than 10 km from the clinic were less likely to follow-up after a positive screening. |
|  |  |  |  |  |  |  |  |  |
|  |  |  |  |  |  | aOR | 95% CI |  |
| Kangmennaang 2015 [44] | Patient-reported lifetime cervical cancer screening | Nationally-representative sample of Namibian women aged 15-64 in 2013 | 6542 | Whether the distance traveled to healthcare was a "big problem" or "not a big problem". | Distance was not a big problem | 0.98 | 0.85 - 1.15 | No relationship between distance as a barrier to receiving healthcare and lifetime cervical cancer screening. |
|  |  |  |  |  |  |  |  |  |
|  |  |  |  |  |  | aOR | 95% CI |  |
| León-Maldonado 2023 [45] | Receipt of follow-up colposcopy after + HPV test | Mexican women aged 25-64 with high-risk HPV-positive results from 2017-2018 | 1339 | Travel time from patient's local healthcare clinic to colposcopy clinic | Travel time (min) | **0.68** | **0.49 - 0.94** | For every minute increase in estimated travel time, the odds of adherence to the colposcopy appointment significantly decreased. |
|  |  |  |  |  |  |  |  |  |
|  |  |  |  |  |  |  |  |  |
| Moss 2022 [46] | Patient-reported Pap test within 3 years or HPV test within 5 years | Convenience sample of English-speaking women aged 50-65 in rural, segregated counties in Pennsylvania in 2019-2020 | 100 | Patient reported travel time to PCP | *No significant results* | | | No relationship between travel time and cervical cancer screening. |
| Pohl 2025 [3] | Patient-reported Pap test within 3 years (21-29) or pap test within 5 years (30-65) | Nationally representative sample of US women age 21-65 | 6756 | Delayed medical care in the past year due to transportation barriers | *No significant results* | | | No relationship between transportation barriers and cervical cancer screening. |
| Sedani 2025 [35] | Screening mammogram within past 2 years | Women aged 21-65 living in the United States who participated in the 2022 Behavioral Risk Factor Surveillance Survey | 56 585 | Lack of reliable transportation | *No significant results* | | | No relationship between lack of reliable transportation and cervical cancer screening. |
| Wilson 2011 [38] | Cervical cancer screening in past 3 years | Randomly selected American-Indian women receiving care at six different Indian Health Service units from 2004-2006 | 900 | Distance to nearest IHS facility | *No significant results* | | | No relationship between cervical cancer screening distance to the closest screening facility. |
| ORs/RRs > 1 indicate increased odds of receiving cancer screening  ^A^ Effect sizes inverted from published results for consistency | | | | | | | | |

Supplemental Table 6. Findings from studies of transportation insecurity and colorectal cancer screening.

| **Study** | **Outcome** | **Population** | **Sample** | **Exposure** | **Results** | | | | **Summary** | |
| --- | --- | --- | --- | --- | --- | --- | --- | --- | --- | --- |
|  |  |  |  |  |  | aOR | | 95% CI |  | |
| Anderson 2013 [47] | FIT w/in 1 year, sigmoidoscopy w/in 5 years, or colonoscopy w/in 10 years (average risk), colonoscopy w/in 5 years (high risk) | Representative sample of adults aged 50-75 living in Utah | 4260 | Travel time by car (min) | > 20 min | **0.64** | | **0.43 - 0.97** | Participants living > 20 minutes from their nearest colonoscopy provider were significantly less likely to be up to date with their colorectal cancer screening than those living within a 10 minute drive. | |
| Aruma 2024 [41] | FIT w/in 1 year, sigmoidoscopy w/in 5 years, or colonoscopy w/in 10 years | Convenience sample of women 45-65 living in Pennsylvania | 474 | Travel time to PCP | *No significant results* | | | | There was no relationship between travel time to PCP and colorectal cancer screening. | |
|  |  |  |  |  |  | aRR | | 95% CI |  | |
| Banegas 2024 [17] | FOBT w/in 1 year, FIT w/in 3 years, sigmoidoscopy or CT colonography w/in 5 years, or colonoscopy w/in 10 years | Adults eligible for colorectal cancer screening in 186 community-based healthcare organizations | 171724 | Patient-reported transportation barriers | Transportation barriers | **0.87** | | **0.83 - 0.90** | Patients who reported transportation barriers had a lower likelihood of being up-to-date on colorectal cancer screening. | |
|  |  |  |  |  |  | aOR | | 95% CI |  | |
| Berthelsen 2022 [48] | Follow-up colonoscopy after +FIT | Adults aged 50-74, living in South-East Norway, who tested + on FIT in an RCT | 3253 | Driving time from patient postal code to screening center |  | **0.86** | | **0.78 - 0.95** | Travel time to cancer center had a significant negative effect colonoscopy completion after positive FIT. | |
|  |  |  |  |  |  |  | |  |  |  |
|  |  |  |  |  |  |  | |  |  |  |
| Chen 2020 [49] | Colonoscopy after +FIT or +screen on high-risk factor questionnaire | Adults aged 50-74 living in Guangzhou, China who screened positive on non-invasive CRC screening (questions or FOBT) | 34606 | Travel time from participants' home to colonoscopy provider using public transit and walking time | *No significant results* | | | | Adherence to colonoscopy decreased with travel time in a univariate model but was not statistically significant in the fully adjusted model. | |
| Franklin 2024 [50] | FOBT/FIT w/in 1 y, stool DNA w/in 3 y, CT colonography w/in 5 y,  flexible sigmoidoscopy w/in 5 y, colonoscopy w/in 10 y | US patients aged 50-74 enrolled in a large HMO | 3443 | Severe transportation needs | *No significant results* | | | | There was no significant relationship between colorectal cancer screening and reporting severe transoportation needs. | |
|  |  |  |  |  |  | aOR | | 95% CI |  | |
| Khoja 2018 [51] | FOBT/FIT in past year or colonoscopy/sigmoidoscopy in past 5 years | Nationally representative sample of adult Saudi Arabians aged 60 and older | 2946 | Who provides your transportation? Options: *Private driver, self, family or friends, public transportation, other.* | myself | **2.52** | | **1.48 - 4.28** | Being able to drive oneself or having a private driver – as compared to relying on a relative - were associated with increased odds of being up to date on colorectal cancer screening. | |
|  |  |  |  |  | private driver | **2.09** | | **1.15 - 3.78** |  |  |
|  |  |  |  |  |  |  | |  |  |  |
| Kupfer 2018 [52] | Adherence to surveillance (inc. physician visits, CEA, colonoscopy) | Post-resection CRC patients at one of the three NCI centers between 2010-2017 | 241 | Distance to treating facility | *No significant results* | | | | No relationship between distance and adherence to overall CRC surveillance; however, lower travel distance was associated with adherence to colonoscopy only (**4.05**, **1.63 – 10.08**). | |
| Moss 2022 [46] | FOBT/FIT in past year or colonoscopy/sigmoidoscopy in past 5 years | Convenience sample of English-speaking women aged 50-65 in rural Pennsylvania counties | 100 | Patient-reported travel time |  |  | |  | No relationship between patient-reported travel time and colorectal cancer screening adherence. | |
|  |  |  |  |  | *No significant results* | | | |  |  |
|  |  |  |  |  |  |  | |  |  |  |
| Ni 2020 [53] | Completion of FIT/FOBT, sigmoidoscopy, or colonoscopy after 1 year | New Yorkers aged 50-75 who were not up to date on CRC screening and who had at least one primary care visit in 2014 within Bellevue Hospital Center | 14425 | Average travel time on public transit from patient's ZIP code |  |  | |  | No significant associations between travel time for walking/public transit and likelihood of receiving any colorectal cancer screening. | |
|  |  |  |  |  | *No significant results* | | | |  |  |
|  |  |  |  |  |  |  | |  |  |  |
|  |  |  |  |  |  | aOR | | 95% CI |  | |
| Peng 2023 [54] | Prior lifetime colorectal cancer screening (any modality) | Convenience sample of adults 45-75 living in rural areas in Alaska, Idaho, Oregon, and Washington | 141 | Sum of answers: *1) access to public transit, 2) access to a caregiver that could drive them to hospital, 3) prohibitive cost of travel to hospital, and 4) travel time > 30 minutes to hospital* | No telehealth | **0.51** | | **0.27 - 0.95** | Higher travel burden was associated with a significant reduction in the odds of having previously undergone colorectal cancer screening in the group with no prior telehealth use, but there was no association between travel burden and prior colorectal cancer screening among telehealth users. | |
|  |  |  | *no telehealth* |  |  |  | |  |  |  |
|  |  |  | 102 |  | telehealth | 1.16 | | 0.57 - 2.40 |  |  |
|  |  |  | *telehealth* |  |  |  |  |  |  |  |
|  |  |  |  |  |  |  | |  |  |  |
| Pohl 2025 [3] | FIT w/in 1 y, stool  DNA w/in 3 y, CT colonography w/in 5 y,  flexible sigmoidoscopy w/in 5 y, colonoscopy within 10 y | Nationally representative sample of US adults age 50-74 | 10736 | Delayed medical care in the past year due to transportation barriers | *No significant results* | | | | No significant relationship between transportation barriers and colorectal cancer screening. | |
|  |  |  |  |  |  | aRR | | 95% CI |  | |
| Sedani 2025 [35] ^A^ | FOBT w/in 1 year, stool DNA w/in 3 years, sigmoidoscopy or CT colonography w/in 5 years, or colonoscopy w/in 10 years | Adults aged 45-75 living in the United States who participated in the 2022 Behavioral Risk Factor Surveillance Survey | 119113 | Lack of reliable transportation | Transportation is a barrier | **0.90** | | **0.86 – 0.94** | Lacking reliable transportation was associated with a decreased likelihood of being up-to-date with colorectal cancer screening. A sensitivity analysis showed that the effect differed by gender: women were more susceptible to the effect of transportation insecurity. | |
|  |  |  |  |  |  | aOR | 95% CI | |  | |
| Vashi 2024 [55] | Return for surveillance after polypectomy within 3 months of recommended interval | Adults due for surveillance colonoscopy after polyp removal | 445 | Travel distance | 10-20 mi | **0.29** | **0.12 - 0.68** | | Living > 10 miles from the facility was negatively associated with returning for surveillance colonoscopy. | |
|  |  |  |  |  | 20-80 mi | **0.35** | **0.13 – 0.93** | |  |  |
|  |  |  |  |  | > 80 mi | **0.26** | **0.10 - 0.67** | |  |  |
|  |  |  |  |  |  | *B* | | SE |  | |
| Vogt 2014 [56] | Percent of individuals who had a colonoscopy within past 4 years | All individuals aged 55+ in the statutory health insurance scheme in Germany with an outpatient visit | 402 districts | Travel time by car (min) | **-0.077** | | | **0.029** | The percent of adults 55+ in each district who had undergone colonoscopy in the past four years was significantly negatively associated with average travel time to colonoscopy provider. | |
|  |  |  |  |  |  |  | |  |  |  |
|  |  |  |  |  |  |  | |  |  |  |
| ORs/RRs > 1 indicate increased odds of receiving cancer screening  ^A^ Effect sizes inverted from published results for consistency | | | | | | | | | |  |

Supplemental Table 7. Findings from studies of transportation insecurity and lung cancer screening.

| **Study** | **Outcome** | **Population** | **Sample** | **Exposure** | **Results** | | | **Summary** |
| --- | --- | --- | --- | --- | --- | --- | --- | --- |
|  |  |  |  |  |  | aOR | 95% CI |  |
| Leishman 2021 [57] | Agreed to lung cancer screening | US Veterans aged 55-80 eligible for lung cancer screening from eight VA medical centers between 2013-2015 | 5551 | Point to point distance | *No significant results* | | | No relationship between distance from the VA facility and agreeing to or attending lung cancer screening. However, veterans with PCPs co-located with a CT scanner were significantly more likely to agree to and receive lung cancer screening. |
|  | Received lung cancer screening |  |  | Point to point distance | *No significant results* | | |  |
|  |  |  |  |  |  | aOR | 95% CI |  |
| Melikam 2023 [58] | Received lung cancer screening | Adults aged 55-77 eligible for lung cancer screening in 2019 in a large, South Carolina health network | 6852 | Driving time to nearest CT scanner | 10-20 min | **0.80** | **0.65 - 0.98** | Patients further from their nearest CT scanner had lower odds of completing lung cancer screening. |
|  |  |  |  |  | > 20 min | **0.68** | **0.54 - 0.85** |  |
| Navuluri 2023 [59] | Received lung cancer screening | Non-Hispanic White or Non-Hispanic Black US Veterans referred for lung cancer screening between 2013-2021 | 4562 | Minimum point-to-point distance to nearest VA CT scanner | *No significant results* | | | No relationship between distance from CT scanner and odds of completing lung cancer screening. |
| Sedani 2025 [35] ^A^ | Low-dose CT w/in past year | Adults aged 50-80 living in the United States who participated in the 2022 Behavioral Risk Factor Surveillance Survey and eligible for lung cancer screening | 7398 | Lack of reliable transportation | *No significant results* | | | No relationship between transportation needs and lung cancer screening; results invariant by gender |
| Yi 2024 [60] | Ever received low-dose CT after physician order | Patients with orders for lung cancer screening at a safety-net hospital in Texas between 2017-2022 | 2287 | Public transportation and private vehicle transit times to their documented CT scanning facility | *No significant results* | | | No relationship between transit time by car or by public transportation and completion of low-dose CT screening. |

Supplemental Table 8. Findings from studies of transportation insecurity and other types of cancer screening.

| **Study** | **Outcome** | **Population** | **Sample** | **Exposure** | **Results** | | | **Summary** |
| --- | --- | --- | --- | --- | --- | --- | --- | --- |
|  |  |  |  |  |  | aOR | 95% CI |  |
| Shepperd 2015 [64] | Attending an oral cancer examination | Black and White men aged 40 and older living in Florida with history of smoking | 315 | Self-reported transportation to appointment | Had transportation | **1.30** | **1.01 - 1.67** | Patients who reported having transportation on the day of their scheduled appointment had higher odds of attending an oral cancer examination. |
|  |  |  |  |  |  | aOR | 95% CI |  |
| Tam 2019 [65] | Adherence to post-treatment surveillance | Head and neck cancer patients with a one-year disease free interval and presenting for follow-up at MD Anderson Cancer Center | 221 | Route distance from patient's residence to cancer center | 101 - 200 mi | **0.36** | **0.16 - 0.81** | Driving distances of > 100 miles from the main campus were associated with significantly decreased odds of adherence to NCCN-recommended post-treatment surveillance. |
|  |  |  |  |  | >200 mi | **0.39** | **0.20 - 0.76** |  |
|  |  |  |  |  |  |  |  |  |
|  |  |  |  | Route distance |  | aOR | 95% CI |  |
| Rodriguez-Villalvazo 2020 [63] | HCC surveillance (AFP + imaging) | US Veterans with cirrhosis with at least six months of follow-up between 2009-2015 | 61770 | To nearest VA hospital | > 60 mi | **0.86** | **0.80 - 0.92** | Distance to medical center was a significant and non-linear predictor of obtaining complete screening with AFP and US. Distance to primary care provider generally had no significant effect. |
|  |  |  |  | To patient's assigned PCP | *No significant results* | | |  |
|  |  |  |  |  |  |  |  |  |
|  |  |  |  |  |  | B | 95% CI |  |
| Augustin 2020 [61] | Skin cancer screenings per 100,000 insured people | German adults who were covered by statutory health insurance in 2015 | 70200921 | Travel time by car to the next major urban center | Skin cancer screenings completed by GPs | **54.2** | **14.3 - 94.2** | There was no effect of travel time on the number of screenings conducted by dermatologists. However, for each additional minute of travel, 54 (95% CI 14-94) additional skin cancer screenings were conducted by GPs per 100,000 people. |
|  |  |  |  |  |  |  |  |  |
| Montgomery 2025 [62] | > = 1 node surveillance ultrasound per 6 months | Patients undergoing sentinel lymph node biopsy for cutaneous melanoma | 519 | Travel distance | *No significant results* | | | There was no association between travel distance and adherence to ultrasound surveillance among patients who had prior sentinel node biopsy for melanoma. |
|  |  |  |  |  |  | B | SE |  |
| Vogt 2014 [56] | Prostate cancer screening | Individuals with state health insurance in Germany with an outpatient visit between 2008-2011 | 402 districts | Travel time  *Prostate cancer*  *Skin cancer* | *No significant results* | | | Skin cancer screenings were significantly negatively associated with travel time, but prostate cancer screening was not. |
|  | Skin cancer screening |  |  |  | Travel time | **-0.186** | **0.089** |  |

Supplemental Table 9. Findings from studies of transportation insecurity and stage at diagnosis in cervical cancer.

| **Study** | **Outcome** | **Population** | **Sample** | **Exposure** | **Results** | | | **Summary** |
| --- | --- | --- | --- | --- | --- | --- | --- | --- |
|  |  |  |  |  |  | aOR | 95% CI |  |
| Friebel-Klingner 2022 [86] | Stage at Diagnosis | Cervical cancer patients referred to one of two tertiary hospitals in Botswana between 2015 - 2020 | 959 | Travel time from patient's village to the multidisciplinary clinic | 3-5 hr | **2.97** | **1.28 - 6.90** | The odds of being diagnosed with stage IV cervical cancer (vs stage I) significantly increased with travel time. |
|  |  |  |  |  | > 5 hr | **4.26** | **1.61 - 11.3** |  |
| Murage 2018 [77] | Diagnosis by emergent presentation | Patients diagnosed with primary cervical cancer in several cancer registries in England between 2006-2010 | 11599 | Travel time to GP | *No significant results* | | | No significant relationship between travel time to GP and emergent presentation of cervical cancer. |
| Virgilsen 2019 [87] | Stage at diagnosis | Patients in the Danish Cancer Register (DCR) diagnosed with cervical cancer between 2005-2016 | 3758 | Route distance between patient and their PCP. | *No significant results* | | | Neither distance to GP nor diagnosing hospital was significantly associated with advanced cervical cancer. |
|  |  |  |  | Route distance between patient and diagnosing facility. | *No significant results* | | |  |

Supplemental Table 10. Findings from studies of transportation insecurity and stage at diagnosis in colorectal cancer.

| **Study** | **Outcome** | **Population** | **Sample** | **Exposure** | **Results** | | | **Summary** |
| --- | --- | --- | --- | --- | --- | --- | --- | --- |
| Charlton 2016 [88] | Stage at diagnosis | Iowa Medicare beneficiaries aged 65-84 diagnosed with colorectal cancer between 2002-2009 | 4710 | Travel time to colonoscopy provider | *No significant results* | | | No association between travel time and stage at diagnosis |
| Crawford 2012 [89] | Stage at diagnosis | All patients diagnosed with rectal or colon cancer between 1994-2002 in the Northern and Yorkshire Cancer Registry | *Rectal*  7058 | Travel time by car from the patient's home to healthcare providers | *No significant results* | | | No effect of travel time on stage at diagnosis in either colon or rectal cancer. No interaction with area deprivation. |
|  |  |  | *Colon*  11163 | Travel time by car from the patient's home to healthcare providers | *No significant results* | | |  |
|  |  |  |  |  |  | aOR | 95% CI |  |
| Massarweh 2014 [90] | Stage at diagnosis | US adults diagnosed with colon adenocarcinoma in the National Cancer Database between 2003-2010 | 296474 | Point to point distance between patient and diagnosing facility | > 50 mi | **1.18** | **1.12 - 1.24** | A distance of >50 miles to the diagnosing/treating facility was associated with an increased odds of metastatic disease at diagnosis after adjustment for potential confounders. |
| Murage 2017 [91] | Stage at diagnosis | People living in northern Scotland in the CRUX database diagnosed with colorectal cancer between 1997-1998 | 926 | Travel time from patient's home postcode to PCP postcode | *No significant results* | | | No association between travel time and stage at diagnosis in the adjusted model, nor in the model that included an interaction term between travel time and urban/rural status. |
|  |  |  |  |  |  | aRR | 95% CI |  |
| Murage 2018 [77] | Diagnosis via emergent presentation | Patients diagnosed with primary colorectal cancer in several cancer registries between 2006-2010 in England | 150054 | Travel time to GP | 20 - 30 min | **1.22** | **1.08 - 1.37** | Patients with colorectal cancer had significantly higher odds of diagnosis with an emergent presentation as travel time to PCP increased. |
|  |  |  |  |  | > 30 min | **1.39** | **1.19 - 1.62** |  |
|  |  |  |  |  |  | aOR | 95% CI |  |
| Schlottmann 2020 [92] | Stage at diagnosis | US adults diagnosed with colorectal cancer registered in the National Cancer Database between 2004-2015 | 361187 | Point to point distance between patient and diagnosing and/or reporting facility | >18 mi | **1.20** | **1.17 - 1.23** | The relationship between colorectal cancer stage at diagnosis and travel distance was variable, with most combinations of distance/stage showing no effect. There were increased odds of being diagnosed at Stage IV at the longest travel distances. |
| Scoggins 2012 [79] | Stage at diagnosis | Adults aged 18-64 enrolled in Medicaid and diagnosed with colorectal cancer in the Washington State Cancer Registry between 1997-2003 | 723 | Travel time to PCP | *No significant results* | | | Increasing travel time/distance traveled was not significantly associated with an increased stage at diagnosis. |
|  |  |  |  |  |  | aOR | 95% CI |  |
| Virgilsen 2019 [87] | Stage at diagnosis | Patients in the Danish Cancer Register (DCR) diagnosed with rectal or colon cancer between 2005-2016 | *Rectal*  12835 | Distance to diagnosing hospital | > 62 km | **1.32** | **1.10 - 1.59** | Distances of >62km between patients and their diagnosing hospital are significantly associated with diagnosis at advanced tumor stage for rectal but not colon cancer. There was no association between distance between patients and their GP and advanced tumor stage at diagnosis for either colon or rectal cancer. |
|  |  |  | *Colon*  25257 | Distance to diagnosing hospital | *No significant results* | | |  |

Supplemental Table 11. Findings from studies of transportation insecurity and stage at diagnosis in head and neck cancer.

| **Study** | **Outcome** | **Population** | **Sample** | **Exposure** | **Results** | | | **Summary** |
| --- | --- | --- | --- | --- | --- | --- | --- | --- |
|  |  |  |  |  |  | aOR | 95% CI |  |
| Farquhar 2019 [93] | Stage at diagnosis | Adult cases of squamous cell head and neck cancer in the North Carolina Central Cancer Registry between 2002-2006. | 808 | Travel time from patient residence to biopsy location | 22-42 min | **1.63** | **1.04 - 2.56** | Patients living further from their biopsy location had a significantly increased odds of presenting with late-stage head and neck cancer. |
|  |  |  |  |  | 42 - 120 min | **2.08** | **1.29 - 3.34** |  |
|  |  |  |  |  |  | aOR | 95% CI |  |
| Morse 2021 [94] | Stage at diagnosis | Americans with primary laryngeal squamous cell carcinoma included in the National Cancer Database between 2004-2016 | 60439 | Point to point distance | 12.5 - 50 mi | **1.07** | **1.02 - 1.12** | Traveling further than 12.5 miles to the reporting facility was significantly associated with increased odds of having an advanced stage at diagnosis. |
|  |  |  |  |  | > 50 mi | **1.26** | **1.17 - 1.36** |  |
|  |  |  |  |  |  | aOR | 95% CI |  |
| ReyesOrozco 2023 [95] | Stage at diagnosis | Americans diagnosed with primary nasopharyngeal cancer in the National Cancer Database between 2004-2018 | 5652 | Point to point distance from patient to reporting facility | 12.5 - 50 mi | **1.29** | **1.13 - 1.48** | Greater distance between the patient's home and reporting hospital was significantly associated with an increased odds of late stage at presentation. |
|  |  |  |  |  | 50 - 250 mi | **1.49** | **1.21 - 1.84** |  |
| Ringstrom 2018 [96] | Stage at diagnosis | Adults with a primary diagnosis of oral or oropharyngeal cancer in the Kentucky Cancer Registry between 2002-2011 | *Oral Cancer* 2415 | Route distance between patient's address and nearest ENT | *No significant results* | | | In patients with oral and oropharyngeal cancer, there was no relationship between distance to an ENT provider and stage at diagnosis. |
|  |  |  | *Oropharyngeal cancer*  1258 |  | *No significant results* | | |  |
| Vasan 2024 [97] | Stage at diagnosis | Americans diagnosed with HPV-positive oropharyngeal squamous cell carcinoma in the National Cancer Database between 2010 – 2019 | *Oropharyngeal*  8061 | Point to point distance from patient to reporting facility | *No significant results* | | | In patients with HPV+ oropharyngeal cancer, there was no relationship between distance from medical facility and stage at diagnosis. |

Supplemental Table 12. Findings from studies of transportation insecurity and stage at diagnosis in lung cancer.

| **Study** | **Outcome** | **Population** | **Sample** | **Exposure** | **Results** | | | **Summary** |
| --- | --- | --- | --- | --- | --- | --- | --- | --- |
|  |  |  |  |  |  | aOR | 95% CI |  |
| Hutchings 2024 [98] | Stage at diagnosis | Michiganders diagnosed with NSCLC from 1985 to 2018 | 141977 | Travel time to nearest medical facility | < 15 minutes | **1.09** | **1.04 – 1.15** | Living closer to the nearest medical facility was associated with an increased stage at diagnosis. |
|  |  |  |  |  |  | aRR |  |  |
| Murage 2018 [77] | Stage at diagnosis | Patients diagnosed with primary lung cancer in several cancer registries in England between 2006-2010 | 157773 | Travel time to GP | 10 - 20 min | **1.09** | **1.05 - 1.14** | Patients had significantly higher odds of diagnosis with an emergent presentation as travel time to GP increased. |
|  |  |  |  |  | 20 - 30 min | **1.13** | **1.01 - 1.27** |  |
| Scoggins 2012 [79] | Stage at diagnosis | Adults aged 18-64 enrolled in Medicaid diagnosed with lung cancer in the Washington State Cancer Registry between 1997-2003 | 1787 | Travel time and distance between patient and PCP zip codes | *No significant results* | | | Increasing travel time/distance traveled was not associated with stage at diagnosis. |
|  |  |  |  |  |  |  | 95% CI |  |
| Tracey 2015 [99] | Stage at diagnosis | Adults diagnosed with non-small cell lung cancer between 2000-2008 in the New South Wales Central Cancer Registry | 22260 | Distance to the nearest public hospital with a thoracic surgery service | 40-99 km | **1.18** | **1.07 - 1.31** | Patients living further from a specialized public hospital with thoracic surgery service had increased odds of presenting with a higher stage at diagnosis. |
|  |  |  |  |  | > 100 km | **1.17** | **1.08 - 1.27** |  |
|  |  |  |  |  |  | aOR |  |  |
| Virgilsen 2019 [87] | Stage at diagnosis | Patients in the Danish Cancer Register (DCR) diagnosed with lung cancer between 2005-2016 | 44318 | Route distance between patient and their PCP. | > 9 km | **0.87** | **0.75 - 0.95** | Patients living greater distances from their GP or diagnosing hospital had a significantly lower odds of being diagnosed with an advanced stage tumor than those living 0-0.5 km away. |
|  |  |  |  | Route distance between patient and diagnosing facility. | 20-62 km | **0.87** | **0.80 - 0.95** |  |
|  |  |  |  |  | > 62 km | **0.64** | **0.57 - 0.71** |  |

Supplemental Table 13. Findings from studies of transportation insecurity and stage at diagnosis in melanoma.

| **Study** | **Outcome** | **Population** | **Sample** | **Exposure** | **Results** | | | **Summary** |
| --- | --- | --- | --- | --- | --- | --- | --- | --- |
|  |  |  |  |  |  |  | 95% CI |  |
| Snyder 2017 [100] | Stage at diagnosis (Breslow Depth) | All adults diagnosed with melanoma at University of Colorado between 2006-2016 | 1156 | Distance from centroid of patient's zip code to medical facility | > 50 mi | **1.58** | **1.24 - 2.01** | Patients traveling more than 50 miles to a tertiary care center had higher odds of having a higher Breslow's Depth at diagnosis. |
|  |  |  |  |  |  | aOR | 95% CI |  |
| Virgilsen 2019 [87] | Stage at diagnosis | Patients in the Danish Cancer Register (DCR) diagnosed with melanoma between 2005-2016 | 19597 | Route distance between patient and diagnosing facility. | > 62 km | **1.35** | **1.08 - 1.68** | There was no association between distance from GP and stage at diagnosis. However, odds of late stage at diagnosis increased with distance to the diagnosing facility. |
|  |  |  |  | Route distance between patient and PCP. | *No significant results* | | |  |

Supplemental Table 14. Findings from studies of transportation insecurity and stage at diagnosis in other cancer types.

| **Study** | **Outcome** | **Population** | **Sample** | **Exposure** | **Results** | | | **Summary** |
| --- | --- | --- | --- | --- | --- | --- | --- | --- |
|  |  |  |  |  |  | aOR | 95% CI |  |
| Desjardins 2024 [101] | Stage at diagnosis | Adults referred to Johns Hopkins Comprehensive Cancer Center from 2010 - 2019 | *Combined*  94007 | Travel distance | 75 – 95 %tile | **0.79** | **0.72 - 0.87** | Distances >= 75^th^ percentile were significantly associated with lower stages at diagnosis among patients at Johns Hopkins; however, when the cohort was stratified by treatment there was reveral of the association between stage at diagnosis and and distance, with those traveling distances >= 75^th^ percentile who received only diagnosis (**1.34, 1.04 – 1.74)** or only treatment (**1.44, 1.28 – 1.61**) having increased stage at diagnosis. |
|  |  |  |  |  | >95%tile | **0.72** | **0.63 - 0.82** |  |
|  |  |  |  |  |  |  |  |  |
| Kumar 2024 [102] | Stage at diagnosis | Adults diagnosed with esophageal, gastric, or colorectal cancer in Florida from 1981 - 2016 | *Combined*  379054 | Travel time to GI | 5 min increase | **1.01** | **1.01 – 1.02** | 5-minute increases in driving time to GI were significantly associated with advanced stage at diagnosis. |
|  |  |  |  |  |  | aOR | 95% CI |  |
| Lopez 2023 [103] | Stage at diagnosis | Americans with papillary or follicular thyroid cancer in the National Cancer Database diagnosed between 2004-2016 | *Thyroid*  89105 | Point to point distance | 50 - 75 %tile | **1.06** | **1.01 - 1.11** | Living further from the reporting facility was significantly associated with an increased odds of advanced stage at diagnosis. |
|  |  |  |  |  | > 75 %tile | **1.09** | **1.04 - 1.14** |  |
|  |  |  |  |  |  | aOR | 95% CI |  |
| Lynch 2017 [104] | Grade and stage at diagnosis | Caucasian patients with prostate cancer in Pennsylvania between 1995-2005 | *Prostate*  77,086 | % public transit use |  | **1.05** | **1.00 – 1.09** | In a hypothesis-generating study of all census tract variables, public transit use and vehicle poverty were independently associated with increased grade/stage at diagnosis in prostate cancer. |
|  |  |  |  | % households without car |  | **1.07** | **1.02 – 1.12** |  |
|  |  |  |  |  |  | aOR | 95% CI |  |
| Macleod 2018 [105] | Stage at diagnosis | Americans diagnosed with testicular cancer between 2004-2014 in the National Cancer Database | 31522 | Point to point distance | > 50 mi | **1.29** | **1.14 – 1.45** | Longer distance to the reporting facility was associated with later stage at diagnosis. |
|  |  |  |  |  |  | aRR | 95% CI |  |
| Murage 2018 [77] | Diagnosis with emergent presentation | Patients diagnosed with primary cancer in several cancer registries in England between 2006-2010 | *Prostate*  160668 | Travel time to GP | 20 - 30 min | **1.24** | **1.05 - 1.45** | Patients had higher odds of diagnosis with an emergent presentation for prostate, stomach, and ovarian cancer, but not brain cancer, as travel time to PCP increased. |
|  |  |  |  |  | > 30 min | **1.68** | **1.36 - 2.07** |  |
|  |  |  | *Stomach*  29898 | Travel time to GP | 20 - 30 min | **1.31** | **1.01 - 1.72** |  |
|  |  |  | *Ovarian*  27435 | Travel time to GP | > 30 min | **1.44** | **1.05 - 1.98** |  |
|  |  |  | *Brain*  18034 | Travel time to GP | *No significant results* | | |  |
|  |  |  |  |  |  | aOR | 95% CI |  |
| Regmi 2024 [106] | Stage at diagnosis | Adults aged > 45 diagnosed with differentiated thyroid cancer between 2000-2021 at the University of Nebraska Medical Center | *Thyroid*  800 | Point to point distance to medical facility | ≥ 50 mi | **2.0** | **1.2 – 3.2** | Living ≥50 miles from the treatment center was associated with advanced stage at diagnosis among adults ≥ 45 years old with differentiated thyroid cancer. |
|  |  |  |  | Driving distance to |  | aOR | 95% CI |  |
| Virgilsen 2019 [87] | Stage at diagnosis | Patients in the Danish Cancer Register (DCR) diagnosed with cancer between 2005-2016 | 2939 (Testicular) | Diagnosing facility | > 62 km | **2.55** | **1.62 - 4.01** |  |
|  |  |  |  | PCP | *No significant results* | | |  |
|  |  |  | 3814 (Esophageal) | Diagnosing facility | *No significant results* | | |  |
|  |  |  |  | PCP | *No significant results* | | |  |
|  |  |  | 35385 (Prostate) | Diagnosing facility | > 62 km | **0.84** | **0.73 - 0.97** |  |
|  |  |  |  | PCP | *No significant results* | | |  |
|  |  |  | 4754 (Stomach) | Diagnosing facility |  | **0.6** | **0.42 - 0.86** |  |
|  |  |  |  | PCP | *No significant results* | | |  |
|  |  |  | 7194 (Pancreatic) | Diagnosing facility | > 62 km | **0.52** | **0.40 - 0.69** |  |
|  |  |  |  | PCP | 0.5 - 2 km | **0.8** | **0.65 - 0.98** |  |
|  |  |  | 4719 (Ovarian) | Diagnosing facility | 20-62 km | **0.69** | **0.55 - 0.88** |  |
|  |  |  |  |  | > 62 km | **0.64** | **0.50 - 0.82** |  |
|  |  |  |  | PCP | *No significant results* | | |  |
|  |  |  |  |  |  | | |  |
| Xu 2019 [107] | Stage at diagnosis | All adults diagnosed with biliary cancer between 2001-2015 in Alberta, Canada | 1610 | Driving time to tertiary care | *No significant results* | | | No relationship between driving time to HPB surgeon and stage at diagnosis in biliary cancer. |

D1: Selection and allocation 1

D2: Comprehensiveness of reporting 1

D3: Classification of exposure 1

D4: Classification of exposure 2

D5: Confounding factors 1

D6: Confounding factors 2

D7: Confounding factors 3

D8: Confounding factors 4

D9: Temporal precedence 1

D10: Temporal precedence 2

D11: Classification of the outcome 1

D12: Classification of the outcome 2

D13: Participant retention 1

D14: Participant retention 2

D15: Statistical conclusion validity

D16: Quality of statistical analyses

Low

Uncertain

High

Not Applicable

Risk of Bias

D1 – D15

Excellent

Average

Poor

Statistical Quality

D16

Supplemental Figure 1. Assessment of retained breast cancer screening studies using JBI tools

D1: Selection and allocation 1

D2: Comprehensiveness of reporting 1

D3: Classification of exposure 1

D4: Classification of exposure 2

D5: Confounding factors 1

D6: Confounding factors 2

D7: Confounding factors 3

D8: Confounding factors 4

D9: Temporal precedence 1

D10: Temporal precedence 2

D11: Classification of the outcome 1

D12: Classification of the outcome 2

D13: Participant retention 1

D14: Participant retention 2

D15: Statistical conclusion validity

D16: Quality of statistical analyses

Low

Uncertain

High

Not Applicable

Risk of Bias

D1 – D15

Excellent

Average

Poor

Statistical Quality

D16

Supplemental Figure 2. Assessment of retained cervical cancer screening studies using JBI tools

D1: Selection and allocation 1

D2: Comprehensiveness of reporting 1

D3: Classification of exposure 1

D4: Classification of exposure 2

D5: Confounding factors 1

D6: Confounding factors 2

D7: Confounding factors 3

D8: Confounding factors 4

D9: Temporal precedence 1

D10: Temporal precedence 2

D11: Classification of the outcome 1

D12: Classification of the outcome 2

D13: Participant retention 1

D14: Participant retention 2

D15: Statistical conclusion validity

D16: Quality of statistical analyses

Low

Uncertain

High

Not Applicable

Risk of Bias

D1 – D15

Excellent

Average

Poor

Statistical Quality

D16

D1: Selection and allocation 1

D2: Comprehensiveness of reporting 1

D3: Classification of exposure 1

D4: Classification of exposure 2

D5: Confounding factors 1

D6: Confounding factors 2

D7: Confounding factors 3

D8: Confounding factors 4

D9: Temporal precedence 1

D10: Temporal precedence 2

D11: Classification of the outcome 1

D12: Classification of the outcome 2

D13: Participant retention 1

D14: Participant retention 2

D15: Statistical conclusion validity

D16: Quality of statistical analyses

Low

Uncertain

High

Not Applicable

Risk of Bias

D1 – D15

Excellent

Average

Poor

Statistical Quality

D16

Supplemental Figure 3. Assessment of retained colorectal cancer screening studies using JBI tools

D1: Selection and allocation 1

D2: Comprehensiveness of reporting 1

D3: Classification of exposure 1

D4: Classification of exposure 2

D5: Confounding factors 1

D6: Confounding factors 2

D7: Confounding factors 3

D8: Confounding factors 4

D9: Temporal precedence 1

D10: Temporal precedence 2

D11: Classification of the outcome 1

D12: Classification of the outcome 2

D13: Participant retention 1

D14: Participant retention 2

D15: Statistical conclusion validity

D16: Quality of statistical analyses

Low

Uncertain

High

Not Applicable

Risk of Bias

D1 – D15

Excellent

Average

Poor

Statistical Quality

D16

Supplemental Figure 4. Assessment of retained lung cancer screening studies using JBI tools

D1: Selection and allocation 1

D2: Comprehensiveness of reporting 1

D3: Classification of exposure 1

D4: Classification of exposure 2

D5: Confounding factors 1

D6: Confounding factors 2

D7: Confounding factors 3

D8: Confounding factors 4

D9: Temporal precedence 1

D10: Temporal precedence 2

D11: Classification of the outcome 1

D12: Classification of the outcome 2

D13: Participant retention 1

D14: Participant retention 2

D15: Statistical conclusion validity

D16: Quality of statistical analyses

Low

Uncertain

High

Not Applicable

Risk of Bias

D1 – D15

Excellent

Average

Poor

Statistical Quality

D16

Supplemental Figure 5. Assessment of retained other cancer screening studies using JBI tools

D1: Selection and allocation 1

D2: Comprehensiveness of reporting 1

D3: Classification of exposure 1

D4: Classification of exposure 2

D5: Confounding factors 1

D6: Confounding factors 2

D7: Confounding factors 3

D8: Confounding factors 4

D9: Temporal precedence 1

D10: Temporal precedence 2

D11: Classification of the outcome 1

D12: Classification of the outcome 2

D13: Participant retention 1

D14: Participant retention 2

D15: Statistical conclusion validity

D16: Quality of statistical analyses

Low

Uncertain

High

Not Applicable

Risk of Bias

D1 – D15

Excellent

Average

Poor

Statistical Quality

D16

Supplemental Figure 6. Assessment of retained breast cancer stage at diagnosis studies using JBI tools

D1: Selection and allocation 1

D2: Comprehensiveness of reporting 1

D3: Classification of exposure 1

D4: Classification of exposure 2

D5: Confounding factors 1

D6: Confounding factors 2

D7: Confounding factors 3

D8: Confounding factors 4

D9: Temporal precedence 1

D10: Temporal precedence 2

D11: Classification of the outcome 1

D12: Classification of the outcome 2

D13: Participant retention 1

D14: Participant retention 2

D15: Statistical conclusion validity

D16: Quality of statistical analyses

Low

Uncertain

High

Not Applicable

Risk of Bias

D1 – D15

Excellent

Average

Poor

Statistical Quality

D16

Supplemental Figure 7. Assessment of retained cervical cancer stage-at-diagnosis studies using JBI tools

D1: Selection and allocation 1

D2: Comprehensiveness of reporting 1

D3: Classification of exposure 1

D4: Classification of exposure 2

D5: Confounding factors 1

D6: Confounding factors 2

D7: Confounding factors 3

D8: Confounding factors 4

D9: Temporal precedence 1

D10: Temporal precedence 2

D11: Classification of the outcome 1

D12: Classification of the outcome 2

D13: Participant retention 1

D14: Participant retention 2

D15: Statistical conclusion validity

D16: Quality of statistical analyses

Low

Uncertain

High

Not Applicable

Risk of Bias

D1 – D15

Excellent

Average

Poor

Statistical Quality

D16

Supplemental Figure 8. Assessment of retained colorectal cancer stage-at-diagnosis studies using JBI tools

D1: Selection and allocation 1

D2: Comprehensiveness of reporting 1

D3: Classification of exposure 1

D4: Classification of exposure 2

D5: Confounding factors 1

D6: Confounding factors 2

D7: Confounding factors 3

D8: Confounding factors 4

D9: Temporal precedence 1

D10: Temporal precedence 2

D11: Classification of the outcome 1

D12: Classification of the outcome 2

D13: Participant retention 1

D14: Participant retention 2

D15: Statistical conclusion validity

D16: Quality of statistical analyses

Low

Uncertain

High

Not Applicable

Risk of Bias

D1 – D15

Excellent

Average

Poor

Statistical Quality

D16

Supplemental Figure 9. Assessment of retained Head and Neck Cancer stage-at-diagnosis studies using JBI tools.

D1: Selection and allocation 1

D2: Comprehensiveness of reporting 1

D3: Classification of exposure 1

D4: Classification of exposure 2

D5: Confounding factors 1

D6: Confounding factors 2

D7: Confounding factors 3

D8: Confounding factors 4

D9: Temporal precedence 1

D10: Temporal precedence 2

D11: Classification of the outcome 1

D12: Classification of the outcome 2

D13: Participant retention 1

D14: Participant retention 2

D15: Statistical conclusion validity

D16: Quality of statistical analyses

Low

Uncertain

High

Not Applicable

Risk of Bias

D1 – D15

Excellent

Average

Poor

Statistical Quality

D16

Supplemental Figure 10. Assessment of retained Lung Cancer stage-at-diagnosis studies using JBI tools.

D1: Selection and allocation 1

D2: Comprehensiveness of reporting 1

D3: Classification of exposure 1

D4: Classification of exposure 2

D5: Confounding factors 1

D6: Confounding factors 2

D7: Confounding factors 3

D8: Confounding factors 4

D9: Temporal precedence 1

D10: Temporal precedence 2

D11: Classification of the outcome 1

D12: Classification of the outcome 2

D13: Participant retention 1

D14: Participant retention 2

D15: Statistical conclusion validity

D16: Quality of statistical analyses

Low

Uncertain

High

Not Applicable

Risk of Bias

D1 – D15

Excellent

Average

Poor

Statistical Quality

D16

Supplemental Figure 11. Assessment of retained melanoma stage at diagnosis studies using JBI tools

D1: Selection and allocation 1

D2: Comprehensiveness of reporting 1

D3: Classification of exposure 1

D4: Classification of exposure 2

D5: Confounding factors 1

D6: Confounding factors 2

D7: Confounding factors 3

D8: Confounding factors 4

D9: Temporal precedence 1

D10: Temporal precedence 2

D11: Classification of the outcome 1

D12: Classification of the outcome 2

D13: Participant retention 1

D14: Participant retention 2

D15: Statistical conclusion validity

D16: Quality of statistical analyses

Low

Uncertain

High

Not Applicable

Risk of Bias

D1 – D15

Excellent

Average

Poor

Statistical Quality

D16

Supplemental Figure 12. Assessment of retained stage-at-diagnosis studies of other cancers using JBI tools.

PRISMA checklist

**Preferred Reporting Items for Systematic reviews and Meta-Analyses extension for Scoping Reviews (PRISMA-ScR) Checklist**

| **SECTION** | **ITEM** | **PRISMA-ScR CHECKLIST ITEM** | **REPORTED ON PAGE #** |
| --- | --- | --- | --- |
| **TITLE** | | | |
| Title | 1 | Identify the report as a scoping review. | 1 |
| **ABSTRACT** | | | |
| Structured summary | 2 | Provide a structured summary that includes (as applicable): background, objectives, eligibility criteria, sources of evidence, charting methods, results, and conclusions that relate to the review questions and objectives. | 2 |
| **INTRODUCTION** | | | |
| Rationale | 3 | Describe the rationale for the review in the context of what is already known. Explain why the review questions/objectives lend themselves to a scoping review approach. | 3, 4 |
| Objectives | 4 | Provide an explicit statement of the questions and objectives being addressed with reference to their key elements (e.g., population or participants, concepts, and context) or other relevant key elements used to conceptualize the review questions and/or objectives. | 4 |
| **METHODS** | | | |
| Protocol and registration | 5 | Indicate whether a review protocol exists; state if and where it can be accessed (e.g., a Web address); and if available, provide registration information, including the registration number. | Protocol was denied; exempt |
| Eligibility criteria | 6 | Specify characteristics of the sources of evidence used as eligibility criteria (e.g., years considered, language, and publication status), and provide a rationale. | 5, Supplemental Table 2 |
| Information sources* | 7 | Describe all information sources in the search (e.g., databases with dates of coverage and contact with authors to identify additional sources), as well as the date the most recent search was executed. | 4, 5, Supplemental table 1 |
| Search | 8 | Present the full electronic search strategy for at least 1 database, including any limits used, such that it could be repeated. | Supplemental table 1 |
| Selection of sources of evidence† | 9 | State the process for selecting sources of evidence (i.e., screening and eligibility) included in the scoping review. | 5, 6, supplemental table 2 |
| Data charting process‡ | 10 | Describe the methods of charting data from the included sources of evidence (e.g., calibrated forms or forms that have been tested by the team before their use, and whether data charting was done independently or in duplicate) and any processes for obtaining and confirming data from investigators. | 5, 6 |
| Data items | 11 | List and define all variables for which data were sought and any assumptions and simplifications made. | 8 |
| Critical appraisal of individual sources of evidence§ | 12 | If done, provide a rationale for conducting a critical appraisal of included sources of evidence; describe the methods used and how this information was used in any data synthesis (if appropriate). | 9 |
| Synthesis of results | 13 | Describe the methods of handling and summarizing the data that were charted. | 9 |
| **RESULTS** | | | |
| Selection of sources of evidence | 14 | Give numbers of sources of evidence screened, assessed for eligibility, and included in the review, with reasons for exclusions at each stage, ideally using a flow diagram. | 7-9, supplemental table 2 |
| Characteristics of sources of evidence | 15 | For each source of evidence, present characteristics for which data were charted and provide the citations. | 6-9, Supplemental table 2 |
| Critical appraisal within sources of evidence | 16 | If done, present data on critical appraisal of included sources of evidence (see item 12). | 28, 29, Supplemental Figs. 1A-E & 2A-G |
| Results of individual sources of evidence | 17 | For each included source of evidence, present the relevant data that were charted that relate to the review questions and objectives. | 8-27, Supplemental Figs. 1A-E & 2A-G |
| Synthesis of results | 18 | Summarize and/or present the charting results as they relate to the review questions and objectives. | 8 - 27 |
| **DISCUSSION** | | | |
| Summary of evidence | 19 | Summarize the main results (including an overview of concepts, themes, and types of evidence available), link to the review questions and objectives, and consider the relevance to key groups. | 29-34 |
| Limitations | 20 | Discuss the limitations of the scoping review process. | 35 |
| Conclusions | 21 | Provide a general interpretation of the results with respect to the review questions and objectives, as well as potential implications and/or next steps. | 35 |
| **FUNDING** | | | |
| Funding | 22 | Describe sources of funding for the included sources of evidence, as well as sources of funding for the scoping review. Describe the role of the funders of the scoping review. | 36 |

JBI = Joanna Briggs Institute; PRISMA-ScR = Preferred Reporting Items for Systematic reviews and Meta-Analyses extension for Scoping Reviews.

* Where *sources of evidence* (see second footnote) are compiled from, such as bibliographic databases, social media platforms, and Web sites.

† A more inclusive/heterogeneous term used to account for the different types of evidence or data sources (e.g., quantitative and/or qualitative research, expert opinion, and policy documents) that may be eligible in a scoping review as opposed to only studies. This is not to be confused with *information sources* (see first footnote).

‡ The frameworks by Arksey and O’Malley (6) and Levac and colleagues (7) and the JBI guidance (4, 5) refer to the process of data extraction in a scoping review as data charting*.*

§ The process of systematically examining research evidence to assess its validity, results, and relevance before using it to inform a decision. This term is used for items 12 and 19 instead of "risk of bias" (which is more applicable to systematic reviews of interventions) to include and acknowledge the various sources of evidence that may be used in a scoping review (e.g., quantitative and/or qualitative research, expert opinion, and policy document).

*From:* Tricco AC, Lillie E, Zarin W, O'Brien KK, Colquhoun H, Levac D, et al. PRISMA Extension for Scoping Reviews (PRISMAScR): Checklist and Explanation. Ann Intern Med. 2018;169:467–473. [doi: 10.7326/M18-0850](http://annals.org/aim/fullarticle/2700389/prisma-extension-scoping-reviews-prisma-scr-checklist-explanation).
